# Biosafe cerium oxide nanozymes protect human pluripotent stem cells and cardiomyocytes from oxidative stress

**DOI:** 10.1186/s12951-024-02383-x

**Published:** 2024-03-26

**Authors:** Chengwen Hang, Mohamed S. Moawad, Zheyi Lin, Huixin Guo, Hui Xiong, Mingshuai Zhang, Renhong Lu, Junyang Liu, Dan Shi, Duanyang Xie, Yi Liu, Dandan Liang, Yi-Han Chen, Jian Yang

**Affiliations:** 1 State Key Laboratory of Cardiology, Shanghai East Hospital, Tongji University School of Medicine, Shanghai, 200120 China; 2 Shanghai Arrhythmia Research Center, Shanghai East Hospital, Tongji University School of Medicine, Shanghai, 200120 China; 3 Department of Cardiology, Shanghai East Hospital, Tongji University School of Medicine, Shanghai, 200120 China; 4 Shanghai Frontiers Center of Nanocatalytic Medicine, Shanghai, 200092 China; 5 Department of Toxicology and Forensic Medicine, Faculty of Veterinary Medicine, Cairo University, Giza, 3725005 Egypt; 6 Department of Pathology and Pathophysiology, Tongji University School of Medicine, Shanghai, 200092 China; 7 Department of Cardiology, The Second Hospital of Shanxi Medical University, Taiyuan, 030001 China; 8 Department of Cell Biology, Tongji University School of Medicine, Shanghai, 200092 China; 9 Research Units of Origin and Regulation of Heart Rhythm, Chinese Academy of Medical Sciences, Shanghai, 200092 China

**Keywords:** Cerium oxide nanozymes, Human embryonic stem cells, Cardiomyocytes, Differentiation, Reactive oxygen species, Doxorubicin-induced cardiotoxicity

## Abstract

**Background:**

Cardiovascular diseases (CVDs) have the highest mortality worldwide. Human pluripotent stem cells (hPSCs) and their cardiomyocyte derivatives (hPSC-CMs) offer a valuable resource for disease modeling, pharmacological screening, and regenerative therapy. While most CVDs are linked to significant over-production of reactive oxygen species (ROS), the effects of current antioxidants targeting excessive ROS are limited. Nanotechnology is a powerful tool to develop antioxidants with improved selectivity, solubility, and bioavailability to prevent or treat various diseases related to oxidative stress. Cerium oxide nanozymes (CeONZs) can effectively scavenge excessive ROS by mimicking the activity of endogenous antioxidant enzymes. This study aimed to assess the nanotoxicity of CeONZs and their potential antioxidant benefits in stressed human embryonic stem cells (hESCs) and their derived cardiomyocytes (hESC-CMs).

**Results:**

CeONZs demonstrated reliable nanosafety and biocompatibility in hESCs and hESC-CMs within a broad range of concentrations. CeONZs exhibited protective effects on the cell viability of hESCs and hESC-CMs by alleviating excessive ROS-induced oxidative stress. Moreover, CeONZs protected hESC-CMs from doxorubicin (DOX)-induced cardiotoxicity and partially ameliorated the insults from DOX in neonatal rat cardiomyocytes (NRCMs). Furthermore, during hESCs culture, CeONZs were found to reduce ROS, decrease apoptosis, and enhance cell survival without affecting their self-renewal and differentiation potential.

**Conclusions:**

CeONZs displayed good safety and biocompatibility, as well as enhanced the cell viability of hESCs and hESC-CMs by shielding them from oxidative damage. These promising results suggest that CeONZs may be crucial, as a safe nanoantioxidant, to potentially improve the therapeutic efficacy of CVDs and be incorporated into regenerative medicine.

**Graphical Abstract:**

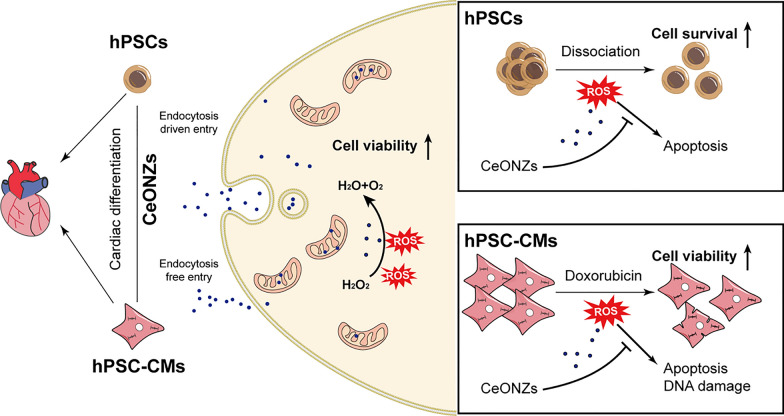

**Supplementary Information:**

The online version contains supplementary material available at 10.1186/s12951-024-02383-x.

## Background

Human pluripotent stem cells (hPSCs), comprising human embryonic stem cells (hESCs) and human induced pluripotent stem cells (hiPSCs), possess the ability to self-renew and differentiate into various cell types [1, 2]. As such, they are valuable tools for both basic research and regenerative medicine [3]. The generation of cardiomyocytes from hPSCs has gained significant interest due to its potential as an important cell source to compensate for the loss of non-renewable cardiomyocytes [4, 5]. However, the culture and differentiation process of hPSCs are susceptible to various factors, such as reactive oxygen species (ROS), which affect the stability of hPSCs and subsequently impact the quality and quantity of their derived cardiomyocytes (hPSC-CMs) [6].

ROS are highly reactive oxygen molecules or oxygen free radicals, involved in numerous physiological and pathological cellular reactions [7]. Studies have demonstrated that ROS can influence self-renewal and differentiation of PSCs [8–11]. A consistent finding regarding the regulation of PSCs by ROS is that at physiological levels, low ROS help maintain genome integrity, while high ROS are necessary for PSCs differentiation [12]. ROS could increase cardiac gene expression and promote myocardial differentiation from hPSCs by activating the Wingless-related integration site (WNT) and Protein kinase B (AKT) signaling pathways [13]. Conversely, abnormal levels of ROS, particularly excessive ROS, can induce oxidative stress, leading to cellular toxicity. Previous studies have shown that elevated ROS could reduce the proliferation and pluripotency of hPSCs [14, 15]. Similarly, treatment with hydrogen peroxide (H_2_O_2_) increased cellular ROS and impaired cardiac differentiation of hESC-derived embryoid bodies (EBs) [16].

Likewise, ROS have dual effects on cardiomyocytes. There is growing evidence that physiological level of ROS facilitated metabolic transition and enhanced contractile force of hPSC-CMs, thereby promoting cardiomyocyte maturation [17]. However, excessive ROS exerted negative impacts on cardiomyocytes, such as calcium homeostasis disruption, arrhythmia, and apoptosis, by stimulating pro-inflammatory cytokines production [18, 19] and were therefore considered common pathological mechanisms underlying various cardiovascular diseases [20, 21]. In addition, high ROS can damage mitochondria, resulting in further ROS production, which contributes to a vicious cycle [22]. It is therefore essential to maintain an appropriate intracellular level of ROS for homeostasis.

Intracellular ROS can be easily elevated by various factors, including changes in culture conditions such as pH, oxygen concentration, and temperature [23]. High enzyme concentration and physical separation during cell passaging also contribute to ROS production [24]. Therefore, it is important to control ROS levels to maintain normal cellular environment during daily culture. To counteract this, traditional antioxidants like vitamins C and E, carotenoids, glutathione, and polyphenols are routinely supplemented in culture media [25]. However, these compounds are heat-sensitive and prone to oxidation [26, 27]. As an alternative, nano-based antioxidants such as silver nanoparticles, cerium oxide nanozymes (CeONZs), graphene oxides, and thiophenol polymers demonstrated more stable and efficient antioxidant effects at the cellular and tissue level [28]. Among these, CeONZs have attracted considerable attention due to their ability to mimic endogenous enzyme activity while overcoming the stability and bioavailability limitations associated with natural enzymes [29]. CeONZs exhibit stable enzyme activity, enabling them to clear ROS at a higher and faster rate than endogenous enzymes. Additionally, they have good biocompatibility and protective effects on various mammalian cells against oxidative stress and inflammation [30–32]. Therefore, CeONZs are promising novel biomaterial in cell culture to enhance cell growth and function.

**Scheme 1:**
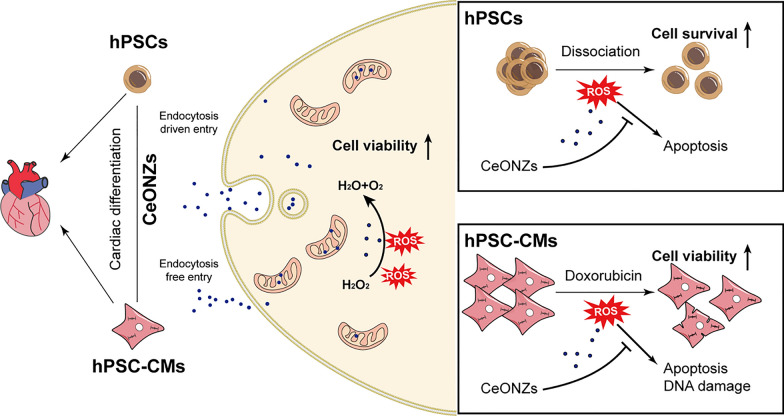
Schematic illustration of CeONZs’ biosafety and applications in hPSCs and hPSC-CMs

In this study, we aimed to provide experimental evidence for the application of CeONZs on human diseases by combining nanotechnology with hPSCs. The biocompatibility, nanosafety, and efficacy of CeONZs in hPSCs and hPSC-CMs were investigated (Scheme 1). The effects of CeONZs on cellular viability, proliferation, and function were examined under both normal and oxidative stress conditions. Moreover, we demonstrated the capacity of CeONZs to protect hPSC-CMs and neonatal rat cardiomyocytes (NRCMs) against doxorubicin-induced cardiotoxicity. Finally, we revealed that CeONZs facilitated diminishing dissociation-induced apoptosis and increased cloning efficiency in hESCs.

## Materials and methods

### Animal

Neonatal Sprague Dawley rats (1–3-day old) were purchased from Shanghai Sippe-Bk Lab Animal Co., Ltd., China. All procedures were performed following the guidelines of Directive 2010/63/EU. All the animal experiments were approved by the Animal Care and Use Committee at Tongji University School of Medicine.

### Chemicals, reagents, and kits

Cerium (III) acetate hydrate (99.9%), ethylene glycol (99.8%), ammonium hydroxide solution (28–30%), sodium azide (NaN_3_), hydrogen peroxide (3%), Triton X-100, bovine serum albumin (BSA), Accutase cell detachment solution, β-mercaptoethanol, paraformaldehyde, dl-Lactate, Gelatin, and 2-phospho-l-ascorbic acid (AA, vitamin C) were purchased from Sigma Aldrich (St. Louis, MA, USA). RPMI 1640 medium, glucose-free RPMI 1640, Phosphate Buffer Saline (PBS; pH 7.2), Penicillin–Streptomycin, serum-free B-27 and B-27 minus insulin supplements, Geltrex, DMEM/F-12, Knockout™ Serum Replacement (KSR), Nonessential amino acids, GlutaMAX, ProLong Gold Antifade Mountant with DAPI, TRIzol reagent, Collagenase II, Mitochondria Isolation Kit were obtained from ThermoFisher Scientific (Waltham, MA, USA). PrimeScript™ RT reagent Kit was acquired from Takara (Kusatsu, Shiga, Japan). ChamQ Universal SYBR qPCR Master Mix was obtained from Nanjing Vazyme Biotech Co., Ltd. (Nanjing, China). Essential 8 medium (E8), EDTA, Cardioeasy I, and II dissociation medium were purchased from Cellapybio Biotechnology Co. Ltd. (Beijing, China). Fetal bovine serum (FBS) was acquired from Excell Biotech Co., Ltd. (Shanghai, China). Y27632 (ROCK inhibitor), Laduviglusib (CHIR-99021), and IWP-2 were obtained from Selleckchem (Houston, TX, USA). Tert-butanol hydrogen peroxide (TBHP) was purchased from Macklin (Shanghai, China). 2-deoxy-d-glucose (2-DG) was acquired from Aladdin Biochemical Technology Co., Ltd. (Shanghai, China). Concentrated Hydrochloric acid (35–37%) and concentrated Nitric Acid (65–58%) were purchased from Sinopharm Chemical Reagent Co., Ltd. (Shanghai, China). In Situ Cell Death Detection Kit was obtained from Roche (Basel, Switzerland). Cell counting kit-8 (CCK-8), Reactive Oxygen Species (ROS) detection kit, Annexin V-FITC staining kit, EdU cell proliferation assay kit using tetramethyl benzamide (Beyoclick EdU-TMB), Comet Assay Kit, and Pluripotent Stem Cell Alkaline Phosphatase Color Development Kit were purchased from Beyotime Biotechnology (Shanghai, China). Superoxide Dismutase (SOD) Activity Assay Kit [Water-soluble tetrazolium salt-1 (WST-1) Method] was bought from Solarbio Science & Technology Co., Ltd. (Beijing, China). Doxorubicin (DOX) and Genistein were purchased from MedChemExpress (Shanghai, China).

### Synthesis of cerium oxide nanozymes (CeONZs)

CeONZs were synthesized using ethylene glycol (EG) to coordinate and cap the Ce^3+^ ions, and ammonium hydroxide was used to precipitate the formed nanoparticles according to Caputo et al. [33] with some modifications. Typically, 10 mL of EG was added into 90 mL of double distilled water in a glass beaker. The mixture was left for 10 min under constant magnetic stirring at 600 rpm, 45 °C for complete homogenization. Then, Cerium (III) acetate hydrate (20 mM) and hydrochloric acid solution (1 M) were added while stirring for 20 min till complete dissolution of cerium salt. Ammonium hydroxide solution (28–30%) was added dropwise until pH reached 9.4. Magnetic stirring continued for another 90 min till the initial color changed from dark purple to yellowish purple. The precipitate was collected by centrifugation at 3000 rpm for 5 min, washed thrice with double distilled water, and then twice with absolute ethanol. Finally, the precipitate was poured into glass Petri dishes and dried overnight at 65 °C. The collected coarse powder was ground into fine powder using an agate mortar and stored in a clean screw-capped glass tube till further use.

### Physical and chemical characterization of CeONZs

The morphology and size of synthesized CeONZs were visualized and determined by JEOL 2100F Transmission Electron Microscope (TEM; JEOL, Tokyo, Japan) and Sigma 300 Field Emission-Scanning Electron Microscope (FE-SEM; Zeiss, Oberkochen, Germany). The crystalline structure of cerium powder was analyzed by X-ray diffraction (XRD) using D2 Phaser X-ray diffractometer system (Bruker instrument, Billerica, MA, USA). Surface chemistry analysis and the existence of both Ce^3+^ and Ce^4+^ ions on the surface of CeONZs were confirmed by using the K-Alpha X-ray Photoelectron spectrometer (XPS) system (ThermoFisher Scientific, Waltham, MA, USA). A Zetasizer Nano ZS90 from Malvern (PANalytical Ltd., Malvern, UK) was employed to measure the zeta potential as well as the hydrodynamic size of CeONZs particles. The structure of synthesized particles and their capping chemistry was confirmed by Fourier Transform Infra-Red (FTIR) spectroscopy using Nicolet iS20 system from ThermoFisher Scientific (Waltham, MA, USA). The Ultraviolet (UV) spectra of CeONZs in different cell culture media were recorded by the UV-3600i plus UV–Vis spectrophotometer system (Shimadzu Corp., Kyoto, Japan).

### Cell culture

H9 hESCs and hESC-CMs (H9-CMs) were cultured in Matrigel-precoated plates (Corning Inc., NY, USA). The hESCs were maintained in E8, while hESC-CMs were grown in RPMI 1640 supplemented with B27 (RB) in a humidified incubator at 37 °C with 5% CO_2_. The hESC-CMs were dissociated with Cardioeasy I and II dissociation buffer, counted, and then cultured in RB. For hESCs culture, the cells were dissociated with Accutase, passaged at a ratio of 1:3–1:6, and cultured in E8 supplemented with ROCK inhibitor Y27632 (10 μM) for the first day, followed by E8 alone thereafter.

### Isolation and culture of neonatal rat cardiomyocytes (NRCMs)

NRCMs were isolated as previously described [34]. The neonatal rat hearts were collected in cold PBS on ice after rapid decapitation, cut into 3–10 mm^2^ tissues, and digested with 0.25% trypsin at 4 °C for 6 h. Then the supernatant was removed and the tissues were digested with collagenase II at 37 °C for 15 min. After standing for several minutes, the supernatant was collected. The digestion with collagenase II was repeated 3 to 4 times until the tissues were not visible. After passing through a 100 μm filter, the supernatant was centrifuged at 1000 rpm for 5 min. The pellet was resuspended in DMEM with 10% FBS. The cell suspension was purified using differential adhesion for 90 min. Finally, the unattached cells were collected and replated into Geltrex-coated dishes for subsequent experiments.

### Differentiation of hESCs into cardiomyocytes

H9 hESCs were differentiated to generate H9-CMs, according to Lian et al. [35] with some modifications. Briefly, hESCs were dissociated at 90% confluency and 3 × 10^5^ cells were plated into 6-well plate in E8 supplemented with Y27632 for the first 24 h. From the second day till the beginning of differentiation, the cells were maintained in plain E8. On the day of induction (D0), the WNT pathway was activated by incubating cells in the induction medium (RPMI 1640 with B27 minus insulin; RB−) supplemented with GSK3β inhibitor, 6 μM Laduviglusib (CHIR-99021), and 0.5 mM AA for 48 h. On day 2 (D2), the cells were grown in RB− with AA only till day 4 (D4). Then, the cells were incubated in RB− supplemented with WNT inhibitor, 5 μM IWP2, and AA for another 2 days. On day 6 (D6), the medium was changed back to RB− and AA only for 48 h. From day 8 (D8), the differentiated cardiomyocytes were maintained in RB, which was replenished every 2 days. Beating cardiomyocytes were monitored starting from day 7 (D7) after induction. Furthermore, the cells were incubated in a glucose-free RB with 4 mM dl-lactate for 4 days to purify cardiomyocytes. All cardiomyocytes used in this research were cultured to differentiation day 30 unless otherwise noted.

### Embryoid body (EB) differentiation

hESCs were rinsed with PBS and digested with 0.5 mM EDTA for 5 min before being resuspended in E8 supplemented with 10 μM Y27632. Then, 1 × 10^6^ cells were seeded into 6-cm low-adherent cell culture dishes on a shaker, at 60 rpm, to form EBs, and Y27632 was removed the next day. E8 was changed daily until day 4. The medium was changed to DMEM/F-12 supplied with 20% Knockout™ Serum Replacement, 100 mM of nonessential amino acids, GlutaMAX, Penicillin–Streptomycin, and 0.1 mM of β-mercaptoethanol and replenished every other day until day 12. The EBs were replated into gelatin-coated 24-well plate for further differentiation. The cells were collected on day 26 for subsequent analysis.

### Superoxide dismutase (SOD) activity assay of CeONZs

The SOD activity was measured by the corresponding kits from Solarbio. The reaction system produces superoxide anion (·O_2_^−^) through xanthine and xanthine oxidase. ·O_2_^−^ reacts with WST-1 to generate a water-soluble yellow substance, which absorbs at 450 nm. SOD eliminates ·O_2_^−^, thereby inhibiting the formation of the yellow substance; the deeper the yellow color of the reaction solution, the lower the SOD activity, and vice versa. To conduct the experiment, different concentrations of CeONZs and the reagents provided in the kit were mixed in 96-well plate according to the manufacturer’s instructions, with a total volume of 200 μL per well. After incubating at 37 °C for 30 min, the absorbance at 450 nm was measured with a microplate reader. The elimination rate of ·O_2_^−^ was calculated based on the absorbance from which the SOD activity was determined. In this system, one unit of SOD enzyme activity is defined as the amount of enzyme that results in 50% inhibition of ·O_2_^−^.

### Uptake and stability of CeONZs in H9 hESCs and H9-CMs

Cells were seeded into Matrigel pre-treated 12-well plate at a density of 1.5 × 10^5^ cells per well. After 2 days, H9-CMs were treated with 60 μM CeONZs in RB while hESCs were incubated with 6 μM CeONZs in E8 to determine the cellular uptake and stability at relatively high and low concentrations of CeONZs. Control cells for both cell types were incubated with their plain media without CeONZs. For cellular uptake, cells were collected at different time points (30 min, 1 h, 2 h, and 24 h) within 1 day. For cellular stability, after 24 h of incubation (D1), CeONZs-containing medium was aspired, and replaced with fresh media. The cells were cultured until day 5 and sampled every other day (D3 and D5). At each time point, cells were washed with PBS before their detachment. For both cellular uptake and stability analysis, the collected cells were resuspended in 5 mL concentrated nitric acid (65–68%) for 3 days in dry place for complete digestion and liberation of uptaken Cerium ions.

For mitochondrial uptake of CeONZs, H9 hESCs and H9-CMs were treated with 60 μM CeONZs for 24 h. After incubation, cells were dissociated, pelleted, and counted. The number of cells was adjusted to 2 × 10^6^ cells per aliquot and pelleted again by centrifugation at 1200 rpm for 5 min. All pellets were treated with the reagents of Mitochondria Isolation Kit following the manufacturer’s instructions. Mitochondria were resuspended in 5 mL concentrated nitric acid (65–68%) for 3 days in dry place for complete digestion and liberation of uptaken Cerium ions. The concentrations of Cerium ions liberated from digested cells were quantified by inductively coupled plasma-mass spectrometry (ICP-MS). Results were normalized and plotted to express the concentration of CeONZs acquired by the mitochondria of 1 × 10^4^ cells.

To investigate the cellular uptake mechanism of CeONZs, H9 hESCs or H9-CMs were divided into four groups. The control group was incubated with E8 or RB plus 60 μM CeONZs for 2 h. The second group was pre-treated with NaN_3_ (10 mM) and 2-DG (50 mM) for 30 min before being exposed to 60 μM CeONZs for 2 h. The third group was incubated with Genistein (200 μM) for 1 h before being exposed to 60 μM CeONZs for 2 h. The fourth group was incubated with Genistein (200 μM) for 30 min, then NaN_3_ (10 mM), 2-DG (50 mM) were supplemented together with Genistein for another 30 min before being exposed to 60 μM CeONZs for 2 h. Cells were then collected and processed following the above cellular uptake protocol.

### Proliferation and viability of H9 and H9-CMs

To test the effect of CeONZs on viability and proliferation of H9 hESCs and H9-CMs, cells were seeded in 48-well plate at a density of 3 × 10^4^ cells per well in their respective media. After 2 days, cells were treated with different concentrations of CeONZs for 24 h.

For viability test, cells were incubated with fresh medium containing 10% of CCK-8 reagent for 2 h before measuring the supernatant at 450 nm wavelength. Cell-free wells containing medium only served as blank control, the absorbance of which was subtracted for background correction.

For proliferation assay, cells were incubated for 24 h with 5-ethynyl-2′-deoxyuridine (EdU) in fresh medium, then proceeded for analysis using tetramethyl benzamide kit according to the manufacturer’s instructions. The absorbance was measured at 370 nm wavelength using ClarioSTAR microplate reader (BMG, Germany). The concentrations of CeONZs were plotted against the relative cell viability and proliferation. All the experiments were repeated three times separately.

### COMET assay

H9 hESCs and H9-CMs were treated with different concentrations of CeONZs for 24 h, with cells treated with 200 μM H_2_O_2_ for 2 h serving as positive controls. According to the manufacturer's instructions, first 1% normal melting point agarose gel was prepared by adding 30 µL of preheated agarose at 45 °C to a microscope slide, then letting it solidify at 4 °C for 10 min. Cells were dissociated into single cells, and after centrifugation at 1200 rpm for 5 min, the cells were resuspended in ice-cold PBS at a density of 0.5–1 × 10^6^ cells/mL. Then, 10 µL of cell suspension was mixed with 75 µL of 0.7% low melting point agarose at 37 °C, adding the mixture dropwise onto the solidified gel, followed by 10 min incubation at 4 °C. Next, the slide was placed in lysis buffer at 4 °C for 2 h and then rinsed with PBS for 3 min. The slide was transferred into electrophoresis buffer at room temperature for 40 min to unwind the DNA, followed by electrophoresis at 25 V for 20 min at 4 °C. After electrophoresis, the slide was neutralized in 0.4 M Tris–HCl three times, 5–10 min each, then incubated with propidium iodide (PI) solution for 10–20 min in the dark. Finally, the slide was washed 3 times with ultra-pure water, covered with a coverslip, and observed under a Leica DMi8 microscope (Wetzlar, Germany). Data were analyzed using CaspLab (CASP) software.

### Apoptosis assay

In Situ Cell Death Detection Kit was used to measure apoptosis by flow cytometry analysis. Briefly, the cells were dissociated, resuspended in PBS, then collected and fixed with 4% paraformaldehyde (PFA) for 15 min. After fixation, cells were permeabilized for 2 min at 4 °C, then washed with PBS. Cells were labeled with terminal deoxynucleotidyl transferase dUTP nick-end labeling (TUNEL) reaction mixture for 1 h at 37 °C and analyzed by CytoFLEX LX flow cytometer (Beckman Coulter, Brea, CA, USA).

Apoptosis was also measured with Annexin V-FITC staining kit using flow cytometry analysis. First, the media containing apoptotic cells were collected in a 15 mL centrifuge tube. Then the adherent cells were washed twice with PBS, dissociated and collected in the aforementioned tube. After centrifugation at 1200 rpm for 5 min, the supernatant was discarded and the cell pellet was suspended in 195 μL of Annexin V-FITC binding solution with 5 μL of Annexin V-FITC dye and gently mixed. The cells were incubated in dark for 20 min at room temperature, centrifuged, and the pellet was resuspended in 400 μL of PBS before measuring with CytoFLEX LX flow cytometer.

### Reactive oxygen species (ROS) analysis

The ROS level was measured using ROS detection kit, following the manufacturer’s protocol. Cells were treated with 10 μM Dichloro-dihydro-fluorescein-diacetate (DCFH-DA) in serum-free culture medium and incubated for 30 min at 37 °C. After incubation, cells were washed thrice with PBS, then imaged with Leica DMi8 fluorescent microscope or measured at 488 nm wavelength with ClarioSTAR microplate reader. The Dichloro-fluorescein (DCF) fluorescence was also detected by flow cytometry under a laser beam of 488 nm wavelength.

### Colony formation assay

H9 hESCs were dissociated into single cells with Accutase and seeded into 6-well plate at a density of 6000 cells per well on Mitomycin C treated mouse embryonic fibroblast (MEF) feeder layer in E8, E8 with Y27632 or E8 with CeONZs for the first day and cultured for another 6 days in plain E8. On day 7, the colonies were assessed using Pluripotent Stem Cell Alkaline Phosphatase Colorimetric Kit. Cells were fixed with 4% PFA, washed with PBS, and then stained with alkaline phosphatase staining solution in dark for 30 min at room temperature. After removing the staining solution and washing cells with distilled water twice, the plates were air-dried and examined under a Leica DMi8 microscope. Colonies with homogenous, heterogenous staining and no staining were classified as undifferentiated, partially differentiated, and fully differentiated, respectively [36].

### Immunofluorescence staining

To determine the expression of pluripotency genes (OCT4, SSEA4, SOX2) in H9 hESCs, cardiac specific markers (cTNT, α-Actinin) in H9-CMs and lineage markers (AFP, TUJ1, FLK1) in differentiating EBs, cells were grown on sterile round coverslips (NEST, China). The cells were washed thrice with PBS before fixation with 4% PFA for 15 min, permeabilized with 0.3% Triton X-100 for 15 min, and blocked with 3% BSA for 30 min at room temperature. Then, the cells were incubated with the primary antibodies overnight at 4 °C, followed by incubation with the secondary antibodies in dark for 1–2 h at room temperature. Finally, the cells were imaged using Leica SP8 confocal microscope (Wetzlar, Germany) after counterstaining the nuclei with ProLong Gold antifade solution containing DAPI. Antibodies used are listed in Additional file 1: Table S1.

### Flow cytometry analysis

To quantify the proportion of cTNT^+^ cardiomyocytes in differentiation day 30 H9-CMs and SSEA4^+^ and NANOG^+^ cells in CeONZs treated hESCs, cells were dissociated and harvested by centrifugation at 1200 rpm for 5 min and washed twice in PBS before fixation, permeabilization, and blocking. Then, the cells were sequentially incubated with the primary and secondary antibodies, and washed with PBS. Before sample loading, cellular aggregates were removed by filtering the cell suspension through a 50 μm nylon filter mesh. The analysis was conducted on CytoFLEX LX flow cytometer, and the fluorescence intensity was quantified by CytoExpert software.

### RNA isolation and quantitative reverse-transcription PCR (qRT-PCR)

To test the effects of CeONZs treatment on H9 hESCs, H9-CMs and NRCMs, we examined the expression of pluripotency genes (*OCT4*, *SOX2*, *NANOG*), lineage marker genes (*HHEX*, *T*, *SOX1*, et al.) in H9 hESCs, cardiac sarcomeric genes (*TNNT2*, *ACTC1*, *MYL2*, et al.) in H9-CMs, apoptosis related genes (*BCL2* and *BAX*) and antioxidant genes (*Nfr2*, *Sod2*, *Gpx1*, et al.) in DOX treated H9-CMs and NRCMs. Total RNA was extracted from about 1 × 10^6^ cells using TRIzol. Complementary DNA (cDNA) was synthesized by reverse transcription of RNA with PrimeScript™ RT reagent Kit. Then, the expression of different genes was analyzed by qRT-PCR using cDNA, 2× SYBR Master Mix, and a panel of oligonucleotide primers as shown in Additional file 1: Table S2. The results were compared to *GAPDH* expression using the 2^−ΔΔCT^ relative expression analysis method.

### Microelectrode arrays (MEA) recording

H9-CMs were dissociated and resuspended in RB at a density of 5 × 10^6^ cells/mL. 8 µL of cell suspension was plated into Matrigel-coated CytoView MEA 24-well plate (Axion BioSystems, Atlanta, GA, USA) for field potential and action potential recording. The environment of MEA device was automatically controlled at 37 °C, 5% CO_2_ to maintain the pH and temperature. The data were acquired using the Maestro Pro multi-well MEA platform (Axion Biosystem, America).

### Cardiomyocyte contractility assay

IonOptix LLC’s contractility/photometry system (IonOptix, Milton, MA) was used to measure the contraction of isolated H9-CMs [37, 38]. Briefly, H9-CMs were seeded onto 35-mm confocal dishes precoated with Geltrex. Then cells were analyzed under the IonOptix system at 37 °C with 5% CO_2_. Single spontaneously contracting cardiomyocyte was selected for recording. Contraction activity was measured based on the pixel point changes at cell edge under ‘Cytomotion’ mode and the magnitude of contraction was assessed with pixel correlation.

### Statistical analysis

All data were presented as means ± SEM. One-way ANOVA with Tukey post-test was the main statistical method used for measuring the significance between two groups and/or multiple test groups. The value of P < 0.05 was regarded as statistically significant and expressed with (*), P < 0.01 with (**), P < 0.001 with (***), and P < 0.0001 with (****).

## Results

### Chemical synthesis and characterization of CeONZs

**Fig. 1:**
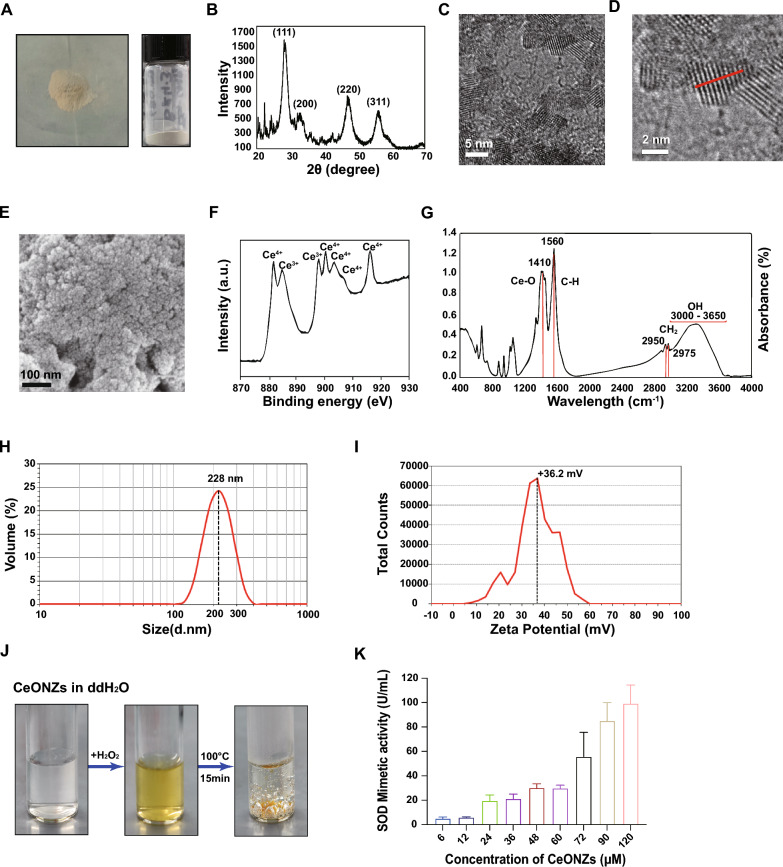
Characterization and in vitro hybrid enzymatic mimetic activity of CeONZs. **A** Appearance of CeONZs powder. **B** XRD pattern of the CeONZs showed four characteristic diffraction peaks. **C** TEM images of CeONZs displayed consistent shape and size with a lattice structure (Scale bar = 5 nm). **D** Magnified TEM image of a single particle where a red line was plotted to calculate the d-spacing of (111) plane (Scale bar = 2 nm). **E** SEM image of small-sized CeONZs (Scale bar = 100 nm). **F** XPS spectrum indicated the presence of Ce^3+^ and Ce^4+^ bivalent states in CeONZs. **G** FTIR spectrum of CeONZs showed the surface capping by ethylene glycol. **H** Hydrodynamic size of CeONZs measured by DLS in ddH_2_O (about 228 nm). **I** Zeta potential of CeONZs in ddH_2_O (+ 36.2 mV). **J** Visual CAT- and SOD-mimetic enzyme activities of CeONZs after H_2_O_2_ addition and boiling for 15 min. **K** Colorimetric SOD-mimetic enzyme activity of different concentrations of CeONZs. Data are presented as mean ± SEM

The chemical precipitation method using ammonium hydroxide was selected to synthesize CeONZs. Cerium (III) acetate hydrate and ethylene glycol were the starter precursors, while the temperature of the chemical reaction was adjusted to 45 °C during the whole process. The formed yellowish-purple precipitate was collected, washed, and dried overnight. The dried coarse powder of CeONZs was ground in an agate mortar and stored in a clean screw-capped glass tube. The color of the final product was pale beige and in the form of fine-textured powder as shown in Fig. 1A.

The ethylene glycol-capped CeONZs (EG-CeONZs) were initially characterized by X-ray diffraction (XRD) and the pattern was shown in Fig. 1B. The resulting data confirmed the cubic fluorite crystalline structure of CeONZs with four characteristic diffraction peaks corresponding to the 111, 200, 220, and 311 planes at 28.1°, 33.1°, 47.1°, and 56.3° respectively as annotated on the diffractogram. The calculated crystalline size of synthesized nanoparticles was 4.5 nm based on Debye–Scherrer’s equation using the position and full width at half maximum of the characteristic (111) diffraction peak. The size and morphology of CeONZs were determined by transmission electron microscope (TEM). Figure 1C showed a mosaic-like pattern of monodisperse nanoparticles with an approximate size of 5 nm. The high-resolution TEM image in Fig. 1D clearly showed the lattice fringes of synthesized nanoparticles, and the d-spacing of the characteristic (111) plane was 0.34 nm as calculated by ImageJ software after plotting a line profile (Red) across the pictured fringes (Additional file 1: Fig. S1A). The small size and spherical morphology of cerium oxide crystallites were confirmed by imaging the nanoparticles using the scanning electron microscope as illustrated in Fig. 1E. The presence of bivalent states of CeONZs was demonstrated in Fig. 1F using the X-ray photoelectron spectroscopy (XPS). The XPS spectrum of cerium ions in CeONZs showed the peaks of Ce^3+^ at binding energies 884.4 and 898.1 eV, while the more abundant peaks of Ce^4+^ were detected at binding energies 881.8, 900.3, 903.3, 906.5, and 915.8 eV. The FTIR data shown in Fig. 1G revealed that the two methylene groups of ethylene glycol were represented by double characteristic bands stretching at 2950 and 2975 cm^−1^, while the stretching of methylene’s C–H atoms was expressed as a single prominent band at 1560 cm^−1^. Also, the O–H stretching of ethylene glycol was shown on the FTIR spectrum as a broad band from 3000 to 3650 cm^−1^. The cerium structure was represented by a single stretching band of Ce–O at 1410 cm^−1^.

The hydrodynamic size of and the charge intensity on CeONZs in aqueous solution and different complete cell culture media were measured by dynamic light scattering (DLS) and zeta potential techniques respectively. The size of CeONZs was ~ 228 nm in double distilled water while it reached ~ 372 and ~ 216 nm when dispersed in E8 and RB, respectively (Fig. 1H and Additional file 1: Fig. S1B). The charge on the surface of CeONZs was + 36.2 mV in distilled water and dropped to ~ − 12 and − 13.2 mV while dispersed in E8 and RB, respectively (Fig. 1I and Additional file 1: Fig. S1C). Moreover, the UV–Vis spectra of different concentrations of CeONZs (300, 600, and 1200 μM) revealed almost identical absorption peaks at 300 nm (Additional file 1: Fig. S1D, E).

As previously reported [39], the synthesized CeONZs can mimic the activity of antioxidant enzymes such as peroxidase and superoxide dismutase, and thus have the ability to scavenge ROS (Additional file 1: Fig. S1F). The catalase (CAT) enzyme-mimicking activity of CeONZs was visualized by adding 50 µL of H_2_O_2_ into 1 mL of colorless aqueous solution of 60 μM dispersed CeONZs. Yellowish color was instantly developed confirming the immediate hydrolysis of H_2_O_2_ by CeONZs. The superoxide dismutase (SOD) mimicking activity of CeONZs was confirmed by reducing the yellowish color to transparent upon boiling for 15 min as shown in Fig. 1J. Furthermore, different concentrations of CeONZs ranging from 6 to 120 μM were tested using a commercial SOD activity detection kit. The colorimetric measurements demonstrated in Fig. 1K revealed the dose-dependent manner of the SOD-mimicking activity (5 to 100 U/mL) of CeONZs, which is in the range of healthy human blood [40–42]. Taken together, ultra-small and spherical CeONZs were successfully synthesized with CAT and SOD mimicking activities.

### Cellular uptake of CeONZs by hESCs and hESC-CMs

**Fig. 2:**
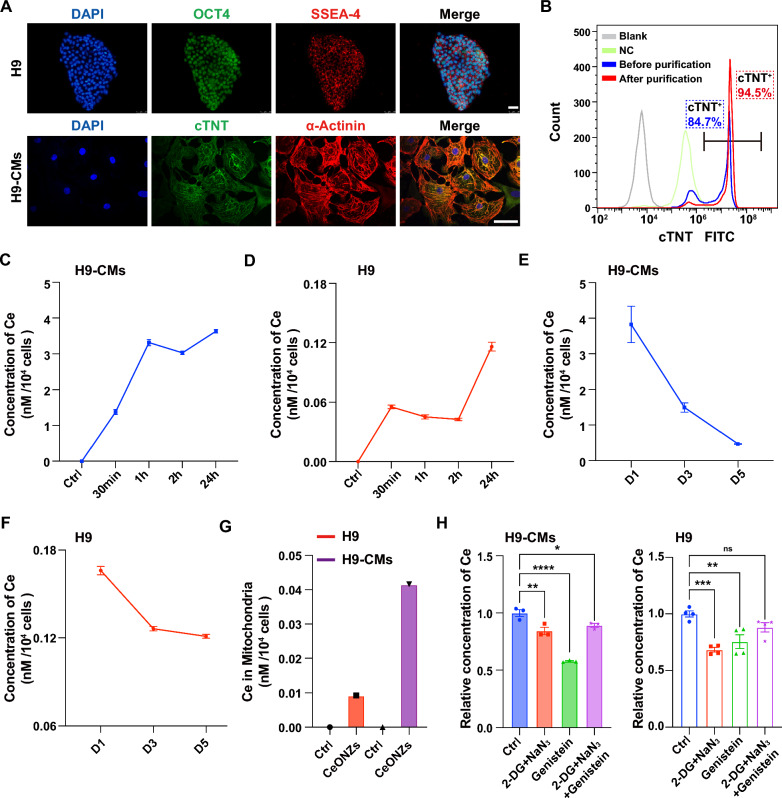
Cellular uptake of CeONZs by hESCs and hESC-CMs. **A** Immunofluorescence analysis revealed the expression of pluripotency markers (OCT4, SSEA-4) in H9 hESCs and CM markers (cTNT, α-Actinin) in H9-CMs (Scale bar = 50 μm). **B** Flow cytometric analysis of the percent of cTNT^+^ CMs on differentiation day 30. The gray curve represents the blank control without staining, the green curve represents the negative control sample stained with the secondary antibody only, and the blue and red curves represent the H9-CMs stained with anti-cTNT antibody before and after purification respectively. **C**, **D** Cellular uptake quantified by cerium concentration per 10,000 cells in H9-CMs (**C**) and H9 hESCs (**D**) exposed to 60 μM and 6 μM CeONZs, respectively, at different time points. **E** CeONZs persistency in H9-CMs, quantified by cerium concentration per 10,000 cells after 24 h exposure to 60 μM CeONZs and further incubation with plain RB medium at days 3 (D3), and 5 (D5) after CeONZs removal. **F** CeONZs persistency in H9 hESCs after 24 h exposure to 6 μM CeONZs and further incubation with plain E8 at D3 and D5 after CeONZs removal. **G** Cerium concentration in mitochondria per 10,000 cells exposed to CeONZs (60 μM) in H9 hESCs and H9-CMs. Ctrl represents H9 hESCs, and H9-CMs without CeONZs treatment. **H** Statistically counted relative cerium concentration in H9 hESCs and H9-CMs co-incubated without drug treatment (Ctrl), with 2-DG and NaN_3_, Genistein, and 2-DG, NaN_3_, together with Genistein, before being exposed to 60 μM CeONZs for 2 h. Data are represented by means ± SEM (n ≥ 3) and statistical significance was determined by one-way ANOVA with a Tukey post-test. *ns* means no significance, *P < 0.05, **P < 0.01, ***P < 0.001, ****P < 0.0001

There is a growing body of evidence suggesting that CeONZs may have protective effects on the cardiovascular system [43–45]. However, it is important to note that most of these experiments have been conducted on animal models, and further research is needed to understand their effects on humans. hPSCs and their derivatives, particularly cardiomyocytes, play a crucial role in modeling human diseases and cardiac regeneration [5, 46]. We differentiated H9 hESCs into cardiomyocytes (H9-CMs), following the protocol outlined in Additional file 1: Fig. S2A. A significant difference in morphology between H9 and H9-CMs was observed (Additional file 1: Fig. S2B). H9 hESCs exhibited high expression of pluripotency markers such as SSEA-4, OCT4, and SOX2, while H9-CMs expressed cardiac-specific markers including cTNT and sarcomere structure genes like α-Actinin (Fig. 2A and Additional file 1: Fig. S2C). Flow cytometry analysis confirmed that the differentiated cardiomyocytes had a high purity of approximately 85% before purification, and increased to 95% after purification (Fig. 2B), thus effectively eliminating the influence of non-cardiomyocytes. The H9-CMs were cultured till differentiation day 30 before analysis.

First, we set to examine the cellular uptake of CeONZs in H9 hESCs and H9-CMs. Upon 60 μM CeONZs treatment, as early as 30 min, CeONZs were present in H9-CMs and gradually accumulated within the cells over 24 h (Fig. 2C). Similar observations were made in H9 hESCs treated with a lower concentration (6 μM) of CeONZs (Fig. 2D). During the first 2 days after the removal of the CeONZs (D3), rapid clearance of the CeONZs was observed, which was followed by a gradual reduction in intracellular CeONZs over the next 2 days (D5) in both H9-CMs and H9 hESCs (Fig. 2E, F). Furthermore, a significant proportion of internalized CeONZs were found to enter the mitochondria, an important organelle for ROS production, in both cell types (Fig. 2G).

In previous studies, CeONZs were found to cross cell membrane through endocytosis and passive cellular uptake [47–49]. To further investigate the mechanism of cellular internalization of CeONZs in human cells, we treated H9-CMs with NaN_3_ and 2-DG to inhibit energy-mediated endocytosis [50] prior to the addition of 60 μM of CeONZs. The results showed that approximately 16% of CeONZs were inhibited from entering the cells through ATP-mediated endocytosis (Fig. 2H). Additionally, when Genistein, an inhibitor of caveolae-mediated endocytosis, was introduced before CeONZs incubation, the cellular phagocytosis efficiency of CeONZs decreased significantly by about 43% (Fig. 2H). Unexpectedly, the combined use of all three drugs inhibited cellular uptake by about 11% (Fig. 2H). Similar results were observed in H9 hESCs, where NaN_3_ combined with 2-DG and Genistein decreased uptake by 32% and 25% respectively, when all three drugs were applied simultaneously, cellular uptake was inhibited by about 12% (Fig. 2H). These findings suggest that ATP and caveolae-mediated cellular endocytosis played a role in the entry of CeONZs into cells, while the cross-talk between these pathways and cross-reactive nature of these inhibitors may affect overall cellular uptake [51].

### CeONZs had favorable bio- and nanosafety in hESCs and hESC-CMs

**Fig. 3:**
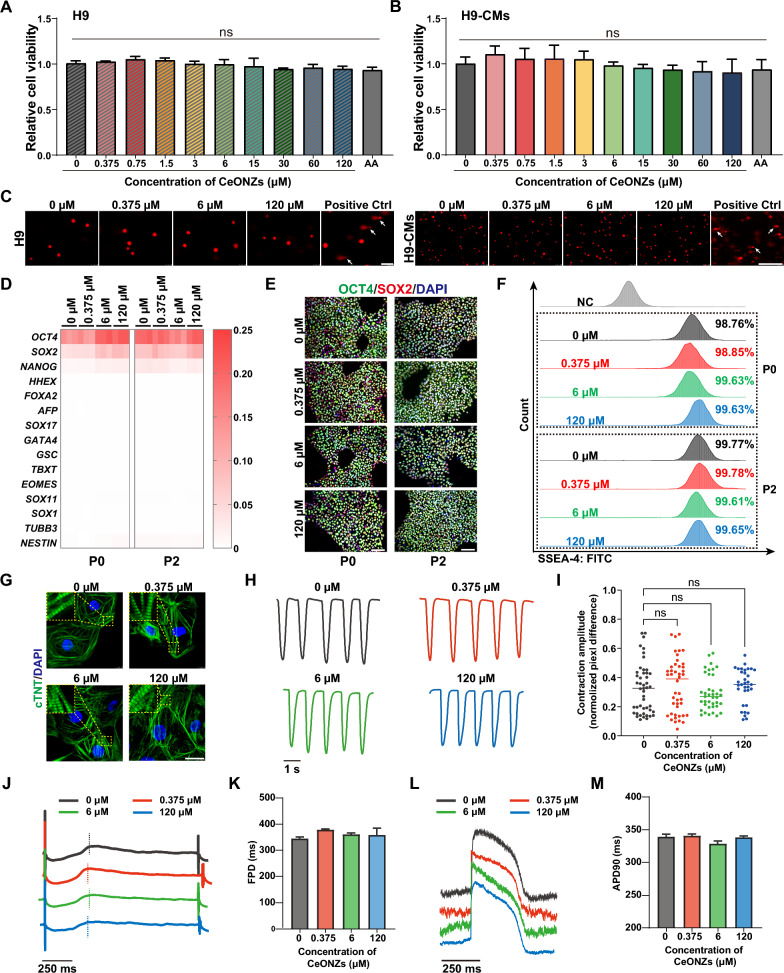
Nanosafety assessment of CeONZs in hESCs and hESC-CMs. **A** and **B** Relative cell viability of H9 hESCs (**A**) and H9-CMs (**B**) after 24 h exposure to varying concentrations of CeONZs (0 to 120 μM) and 0.5 mM AA. **C** Comet assay of H9 hESCs and H9-CMs treated with different concentrations of CeONZs for 24 h, positive control means cells treated with 200 μM H_2_O_2_ for 2 h (Scale bar = 100 μm). Arrows indicate damaged cells. **D** Heatmap of pluripotency and lineage markers expression in H9 hESCs treated with CeONZs for 24 h (Passage 0, P0) and passaged 2 times after CeONZs withdrawal (Passage 2, P2). Relative to *GAPDH* expression (n = 3). **E** Immunofluorescence staining of pluripotency markers OCT4 and SOX2 in CeONZs treated H9 hESCs at P0 and P2 (Scale bar = 100 μm). **F** SSEA-4^+^ cells in CeONZs treated H9 hESCs at P0 and P2. **G** Immunofluorescence staining of cTNT in H9-CMs after 24 h exposure to CeONZs (Scale bar = 25 μm). **H**, **I** Contraction curve (**H**) and contraction amplitude (**I**) of H9-CMs after 1 day of CeONZs treatment (n ≥ 30). **J**–**M** Electrophysiological characteristics of H9-CMs determined by field potential morphology (**J**), field potential duration (FPD) (**K**), action potential morphology (**L**), and action potential duration (APD) (**M**), H9-CMs were treated with low (0.375 μM), medium (6 μM), and high (120 μM) concentrations of CeONZs for 24 h before MEA measurement. Data are presented as means ± SEM (n ≥ 3) and statistical significance was determined by one-way ANOVA with a Tukey post-test. ns means no significance

First, to determine the effects of CeONZs on cell viability, both H9 hESCs and H9-CMs were treated with various concentrations (ranging from 0.375 to 120 μM) of CeONZs for 24 h. The CCK8 assay demonstrated that the cell viability of both cell types was not affected when compared with non-treated or AA-treated cells (Fig. 3A, B). Since the primary target of CeONZs is ROS, we then investigated the impact of CeONZs on cellular ROS levels. Our findings revealed that incubation of H9 hESCs and H9-CMs with different concentrations of CeONZs for 24 h did not alter cellular ROS levels (Additional file 1: Fig. S3A, B). H9 hESCs are pluripotent stem cells that can self-renew, while H9-CMs are similar to human embryonic cardiomyocytes, displaying certain proliferative capacity [52, 53]. We therefore performed EdU incorporation assay to investigate the proliferation of these two cell types in the presence of CeONZs. As shown in Additional file 1: Fig. S3C, D, within the range of concentrations tested, the addition of CeONZs did not significantly affect the proliferation of both H9 hESCs and H9-CMs. Based on this, we choose three representative concentrations, 0.375 μM (low), 6 μM (medium) and 120 μM (high) for further validation.

We then checked the genotoxicity of CeONZs against H9 hESCs and H9-CMs by the comet assay and found that CeONZs did not cause DNA damage in cells at the concentrations of 0.375 μM, 6 μM and 120 μM (Fig. 3C, Additional file 1: Tables S3, S4). To examine the effects of CeONZs on the pluripotency of H9 hESCs, we determined the expression of pluripotency genes (*OCT4*, *SOX2*, and *NANOG*) and lineage marker genes (*TBXT*, *HHEX, SOX1*, et al.) which was comparable among non-treated and CeONZs treated groups at passage 0 (1 day exposure to CeONZs) and passage 2 (consecutively passaged two times after CeONZs removal) (Fig. 3D). Also, immunofluorescence staining of pluripotency markers OCT4 and SOX2 showed no difference (Fig. 3E). The SSEA-4^+^ and NANOG^+^ cells were consistently above 98% in all groups (Fig. 3F, Additional file 1: Fig. S3E). Moreover, the cell viability and intracellular ROS of H9 hESCs were also not affected 2 passages after CeONZs treatment (Additional file 1: Fig. S3F, G).

The internal structure, especially the sarcomere of cardiomyocytes is the base for contraction and relaxation [54, 55]. Therefore, we determined the expression of structurally related genes in H9-CMs exposed to CeONZs for 1 day. Genes involved in cardiac sarcomere structure, such as *ACTC1*, *TNNT2*, *MYL2*, *MYL7*, *MYH6*, *MYH7*, *TNNI1*, and *TNNI3*, did not show significant changes at the transcriptional level (Additional file 1: Fig. S3H). By immunofluorescence staining of sarcomeric-specific marker cTNT, the normal arrangement of sarcomere was observed among all groups (Fig. 3G). Also, we further examined the contractility of cardiomyocytes and found that the contraction amplitude of cardiomyocytes treated with different concentrations of CeONZs for 1 day (D1) was similar to that of the control group (Fig. 3H, I) and did not change significantly when continuously cultured for 3 days after CeONZs withdrawal (D4) (Additional file 1: Fig. S3I). Cardiomyocytes display spontaneous electrical activity, and their contraction and diastole are induced by electro-mechanical coupling. The electrophysiological properties of H9-CMs treated with CeONZs were investigated using MEA to record the field potential (FP) and action potential (AP) (Additional file 1: Fig. S3J, K). First, no significant difference in beat rate was observed in H9-CMs treated with different concentrations of CeONZs at D1 (Additional file 1: Fig. S3L) and D4 (Additional file 1: Fig. S3N). The field potential duration (FPD) after CeONZs treatment was similar to that of the untreated H9-CMs at D1 (Fig. 3J, K) and D4 (Additional file 1: Fig. S3O) too. Concomitantly, the action potential duration (APD) measured by APD90 and APD50 was not significantly different among groups at D1 (Fig. 3L, M and Additional file 1: Fig. S3M). These results showed that CeONZs treatment did not affect the electrophysiological activity of H9-CMs.

### Efficacy of CeONZs in scavenging ROS induced by hydrogen peroxide

Excessive ROS could impair hESCs and their derived cardiomyocytes [56]. To investigate the effectiveness of CeONZs as an antioxidant in protecting H9 hESCs and H9-CMs from stress-induced damage, we used H_2_O_2_ and its derivative *Tert*-butanol hydrogen peroxide (TBHP) as ROS inducers to establish models of oxidative injury on human cells. The results demonstrated that exposing H9-CMs to different concentrations of H_2_O_2_ (ranging from 100 to 400 μM) for 2 h significantly reduced cell viability in a concentration-dependent manner. Cell viability declined by approximately 45% when treated with 200 μM H_2_O_2_. Similarly, lower concentrations of H_2_O_2_ (ranging from 25 to 100 μM) treatment for 24 h also led to concentration-dependent reduction in cell viability, with around 60% decrease in the 100 μM group. Therefore, we selected 200 μM H_2_O_2_ for 2 h and 100 μM H_2_O_2_ for 24 h in subsequent experiments (Additional file 1: Fig. S4A).

**Fig. 4:**
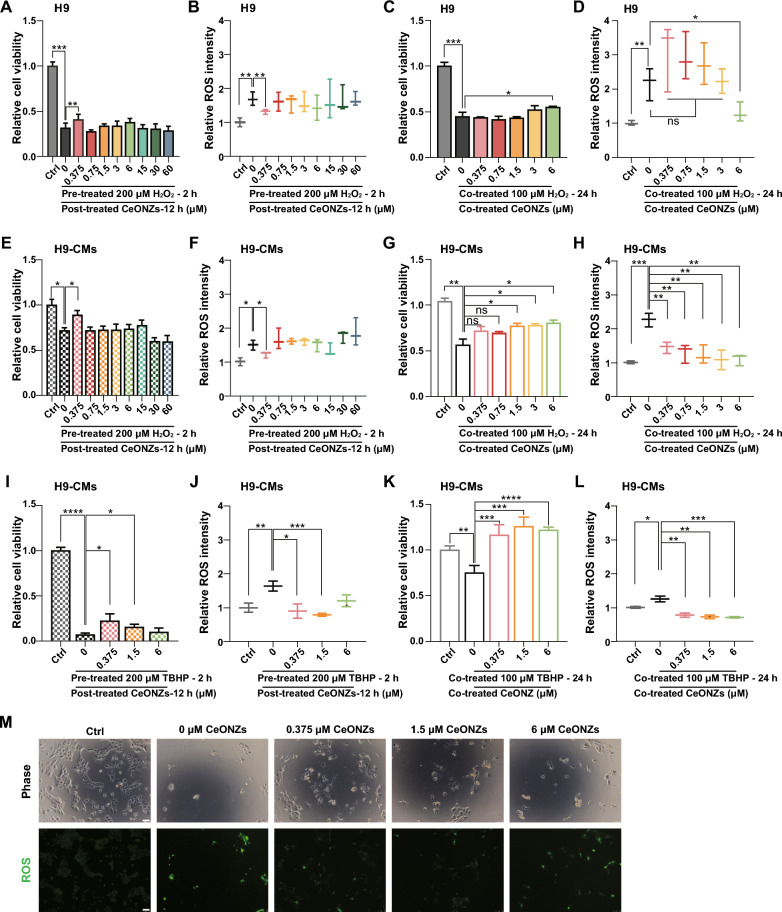
CeONZs protected against H_2_O_2_ and TBHP-induced cell death by reducing excessive ROS. **A**, **B** Relative cell viability (**A**) and ROS intensity (**B**) of H9 hESCs pre-treated with 200 μM H_2_O_2_ for 2 h followed by exposure to CeONZs (0 to 60 μM) for 12 h. Ctrl refers to non-treated cells. **C**, **D** Relative cell viability (**C**) and ROS intensity (**D**) of H9 hESCs co-treated with 100 μM H_2_O_2_ plus CeONZs (0 to 6 μM) for 24 h. **E**, **F** Relative cell viability (**E**) and ROS intensity (**F**) of H9-CMs pre-treated with 200 μM H_2_O_2_ for 2 h, then exposed to CeONZs (0 to 60 μM) for 12 h. Ctrl means non-treated cells. **G**, **H** Relative cell viability (**G**) and ROS intensity (**H**) of H9-CMs co-treated with 100 μM H_2_O_2_ plus CeONZs (0 to 6 μM) for 24 h. **I**, **J** Relative cell viability (**I**) and ROS intensity (**J**) of H9-CMs pre-treated with 200 μM TBHP for 2 h, then exposed to CeONZs (0, 0.375, 1.5 and 6 μM) for 12 h. Ctrl means non-treated cells. **K**, **L** Relative cell viability (**K**) and ROS intensity (**L**) of H9-CMs co-treated with 100 μM TBHP plus CeONZs (0 to 6 μM) for 24 h. **M** Phase and fluorescence images showing ROS of H9-CMs pre-treated with 200 μM TBHP for 2 h, followed by exposure to CeONZs for 12 h (Scale bar = 75 μm). Data are presented as means ± SEM (n ≥ 3) and statistical significance was determined by one-way ANOVA with a Tukey post-test. ns means no significance, *P < 0.05, **P < 0.01, ***P < 0.001, ****P < 0.0001

H9 hESCs were stressed with 200 μM H_2_O_2_ for 2 h, then exposed to plain medium (0 µM) or different concentrations of CeONZs for 12 h. After 14 h of incubation, the viability of cells in plain medium (0 µM CeONZs) dropped to only 30% of the cells without H_2_O_2_ insult. However, when the cells were treated with 0.375 μM CeONZs, their viability was higher than those without CeONZs treatment and was approximately 40% of the cells without H_2_O_2_ insult (Fig. 4A). Analysis of ROS level also revealed that the cells treated with 0.375 μM CeONZs exhibited lower ROS than the 0 µM CeONZs group (Fig. 4B).

We then conducted further investigations on H9 hESCs by co-treating them with 100 μM H_2_O_2_ and CeONZs for 24 h. The H_2_O_2_-treated group showed 55% decrease in cell viability and twofold increase in ROS compared to the untreated control. In contrast, cells co-treated with H_2_O_2_ and 6 μM CeONZs demonstrated 45% decrease in cell viability and 1.3-fold increase in ROS compared to the untreated control (Fig. 4C, D).

Similar experiments were conducted on H9-CMs. After 2 h of 200 μM H_2_O_2_ stress followed by 12 h culture with or without CeONZs, 70% of H9-CMs in plain medium were viable with 1.5-fold increase in ROS compared to the cells without H_2_O_2_ insult. Conversely, in the presence of 0.375 μM CeONZs, the cell viability was boosted to approximately 90%, and ROS decreased to 1.2 times that of the cells without H_2_O_2_ insult (Fig. 4E, F). Furthermore, co-treatment with different concentrations of CeONZs (1.5 μM, 3 μM, and 6 μM) mitigated the decreased cell viability and increased ROS induced by 100 μM H_2_O_2_ for 24 h in H9-CMs (Fig. 4G, H).

To further interrogate the protective effect of CeONZs on human cardiomyocytes, we utilized another oxidant called TBHP. H9-CMs were exposed to various concentrations of TBHP for different durations. The results showed that 200 μM TBHP for 2 h and 100 μM TBHP for 24 h treatments reduced cell viability by 35% and 50%, respectively (Additional file 1: Fig. S4B). Thus, these two settings were selected for subsequent experiments. In H_2_O_2_ stressed model, CeONZs displayed protective effects within the range from 0.375 to 6 μM (Fig. 4A, C, E, G). Based on this finding, we selected 0.375 μM, 1.5 μM, and 6 μM CeONZs for TBHP-induced oxidative damage. After 2 h of 200 μM TBHP treatment followed by 12 h in plain medium without CeONZs, the cell viability declined to only 7% of the untreated control. However, the decline was mitigated by the addition of 0.375 μM and 1.5 μM CeONZs, which maintained cell viability at approximately 22% and 15% of the untreated control respectively (Fig. 4I). Concomitantly, 12 h incubation in plain medium after TBHP removal led to 1.6-fold increase in ROS. In contrast, 0.375 μM and 1.5 μM CeONZs treated cells exhibited low ROS, similar to that of the untreated control (Fig. 4J, M). Finally, the H9-CMs co-treated with TBHP and CeONZs showed more protective effects. When 100 μM TBHP was applied alone for 24 h, cell viability decreased by 30%. In the presence of 0.375 μM, 1.5 μM, and 6 μM CeONZs along with TBHP, the cell viability increased, even surpassing that of the untreated control (Fig. 4K). Accordingly, CeONZs significantly reduced ROS compared to the cells treated with TBHP alone (Fig. 4L). Taken together, our findings demonstrated the protective effects of CeONZs against oxidative stress-induced damage by H_2_O_2_ and TBHP.

### CeONZs protected against DOX-induced cardiotoxicity by scavenging ROS

**Fig. 5:**
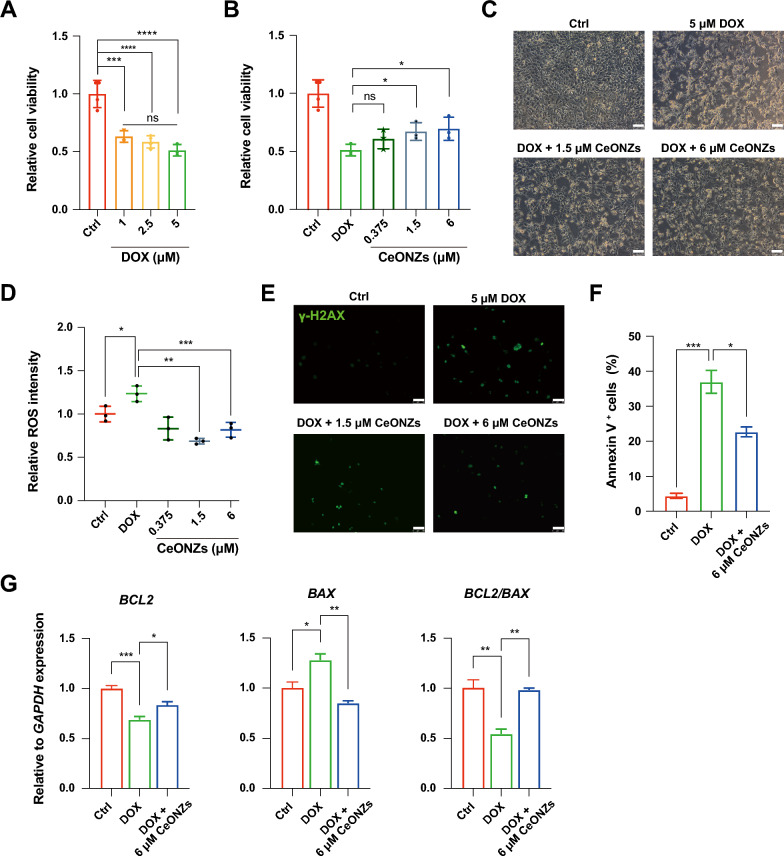
CeONZs mitigated DOX-induced cardiotoxicity in human cardiomyocytes. **A** Cell viability was determined by CCK8 in H9-CMs treated with 1, 2.5, and 5 μM of doxorubicin (DOX) for 24 h. Ctrl refers to non-treated cells. **B** Cell viability of H9-CMs treated with 5 μM DOX in the absence and presence of 0.375, 1.5, and 6 μM CeONZs. **C** Cell morphology of H9-CMs treated with 5 μM DOX in the absence and presence of 1.5, and 6 μM CeONZs (Scale bar = 100 μm). **D** Relative ROS intensity of H9-CMs treated with 5 μM DOX in the absence or presence of different concentrations of CeONZs. **E** Immunofluorescence staining of DNA damage marker γ-H2AX in H9-CMs treated with 5 μM DOX only, 5 μM DOX with 1.5 or 6 µM CeONZs (Scale bar = 50 μm). **F** Percentage of apoptotic H9-CMs treated with DOX alone or in combination with 6 μM CeONZs. Results were determined by percentage of Annexin V^+^ cells. **G** Expression of *BCL2, BAX,* and the ratio of *BCL2/BAX* in H9-CMs treated with DOX or DOX plus 6 μM CeONZs. Relative to *GAPDH* expression. Data are shown as means ± SEM (n = 3) and statistical significance was determined by one-way ANOVA with a Tukey post-test. ns means no significance, *P < 0.05, **P < 0.01, ***P < 0.001, ****P < 0.0001

Anthracyclines are commonly used in chemotherapy to treat cancers [57]. However, one of the most widely used anthracyclines, adriamycin (also known as Doxorubicin or DOX), is known to cause cardiotoxicity due to ROS overproduction [58, 59]. Currently, preventing DOX-induced cardiotoxicity (DOXIC) with antioxidants is not satisfactory and may even cause side effects [60]. To explore the potential of applying CeONZs as ROS scavengers to alleviate DOXIC, we established a DOXIC model by treating differentiation day 120 H9-CMs which were relatively mature and more closely resembled the adult CMs [61, 62] with different concentrations (1, 2.5, and 5 μM) of DOX for 24 h, which caused low cell viability with no statistical difference among the concentrations of DOX tested (Fig. 5A). We then chose 5 μM DOX for subsequent experiments. H9-CMs were co-treated with CeONZs (0.375, 1.5, and 6 μM) and 5 μM DOX for 24 h. We found that H9-CMs co-treated with 1.5 μM and 6 μM CeONZs exhibited a significant increase in cell viability (Fig. 5B, C) and decreased ROS (Fig. 5D). Similar protective effects of CeONZs were observed in differentiation day 30 DOX-treated H9-CMs, while 0.375, and 1.5 μM CeONZs had been more effective in increasing cell viability and decreasing ROS (Additional file 1: Fig. S5A). Also, γ-H2AX immunofluorescence reflecting DNA damage indicated that 5 μM DOX increased DNA damage, which was attenuated by co-treatment with 1.5 μM and 6 μM CeONZs (Fig. 5E). Furthermore, significantly increased Annexin V^+^ cells were detected in DOX-treated H9-CMs, implying extensive apoptosis (Fig. 5F and Additional file 1: Fig. S5B). In contrast, CeONZs decreased the proportion of Annexin V^+^ cells and upregulated anti-apoptosis gene expression (Fig. 5F, G and Additional file 1: Fig. S5B).

To further validate these results, we tested the effect of CeONZs on NRCMs DOXIC model. As expected, the low concentration (0.375, 1.5, and 6 μM) of CeONZs did not affect cell viability (Additional file 1: Fig. S5C). Cell viability of NRCMs treated with different concentrations (1, 2.5, 5, and 10 μM) of DOX for 24 h was decreased but without statistical difference (Additional file 1: Fig. S5D), hence, 5 μM DOX was chosen for subsequent experiments. Consequently, 0.375 μM CeONZs protected NRCMs from DOX-induced cell death (Additional file 1: Fig. S5E), though the DNA damage evaluated by γ-H2AX was not attenuated (Additional file 1: Fig. S5F). However, the expression of anti-apoptosis genes (Additional file 1: Fig. S5G) and antioxidant genes (Additional file 1: Fig. S5H) such as *Nfr2*, *Sod2*, *Gpx1*, and *Gpx4* were upregulated in cells treated with 0.375 μM CeONZs, indicating that CeONZs partially protected DOX-induced NRCMs damage by apoptosis inhibition.

### CeONZs improved cell survival during hESCs maintenance

**Fig. 6:**
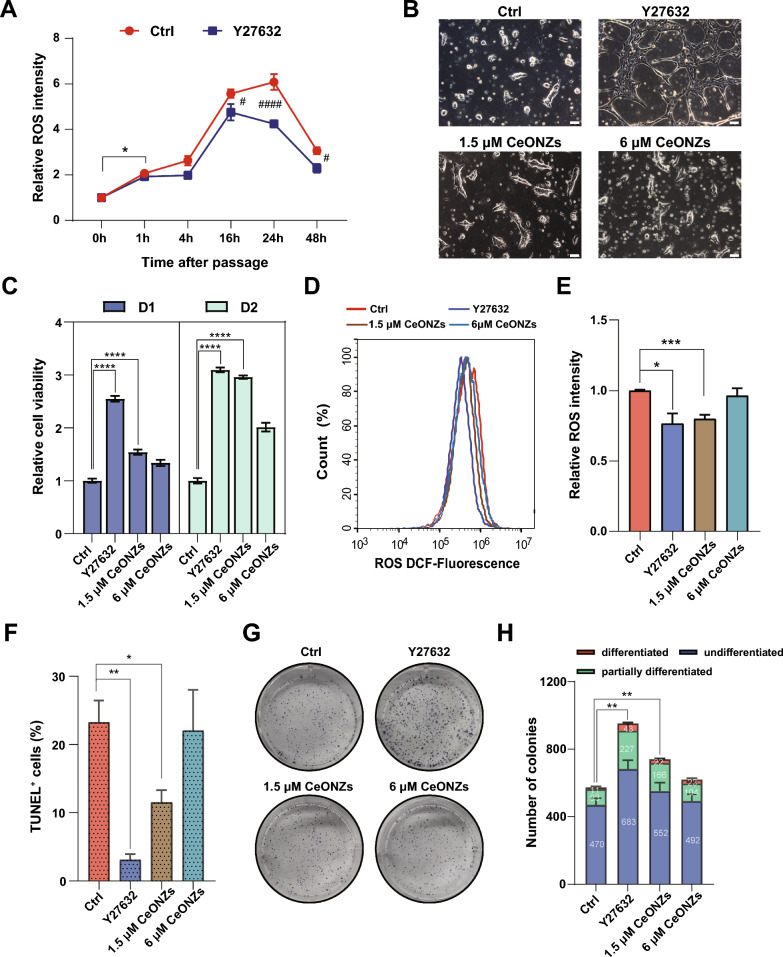
CeONZs permitted survival of dissociated hESCs. **A** Temporal analysis of ROS intensity in H9 hESCs passaged with E8 only and E8 plus Y27632. *Means 1 h vs 0 h in E8 only, ^#^means E8 plus Y27632 vs E8 only. **B** Phase images of H9 cultured in E8 only, E8 plus Y27632, E8 plus 1.5 μM or 6 μM CeONZs for 24 h after cell dissociation (Scale bar = 75 μm). **C** Cell viability of H9 hESCs after seeding 1 day and 2 days. H9 cells were cultured in E8 only, E8 plus Y27632, and E8 plus 1.5 μM or 6 μM CeONZs for 24 h. **D**, **E** Flow cytometry analysis showing ROS levels of H9 cultured in E8 only, E8 plus Y27632, E8 plus 1.5 μM, or 6 μM CeONZs for 24 h after dissociation. **F** TUNEL assay of apoptotic H9 hESCs treated with E8, E8 plus Y27632, or E8 plus indicated concentrations of CeONZs for 24 h after dissociation. **G**, **H** Representative alkaline phosphatase (ALP) staining of H9 hESCs passaged using E8 only, E8 plus Y27632, and E8 plus 1.5 μM or 6 μM CeONZs. The number of colonies was counted on day 7 after plating. *Means the comparison of the total number of colonies. Data are depicted as means ± SEM (n = 3), and statistical significance was determined by one-way ANOVA followed by Tukey’s post hoc test. *P < 0.05, **P < 0.01, ***P < 0.001, ****P < 0.0001. ^#^P < 0.05,  ^####^P < 0.0001

The survival rate of hESCs decreased after dissociation and reinoculation, particularly in serum-free stem cell medium like E8 [63, 64]. To improve cell survival and reduce apoptosis, Y27632, a ROCK inhibitor, was added during passaging [65]. Apoptosis, necrosis, and other cellular processes were affected by excessive ROS [66, 67], leading us to speculate that changes in ROS might be associated with cell survival during passaging. We first monitored ROS levels within 48 h after passaging hESCs with E8 only, which showed that ROS increased significantly 1 h after passaging and continued to rise until 24 h, then began to decrease. However, when E8 was supplemented with Y27632, except 1 h after passaging, ROS was significantly lower at 16 h, 24 h, and 48 h compared to E8 only (Fig. 6A). These data implied that ROS could be a critical factor affecting cell survival during hESCs culture. Thus, we applied CeONZs during the passaging to investigate whether hESCs could be protected from ROS insults. 1.5 μM CeONZs displayed beneficial effects (Fig. 6B), which was confirmed by CCK8 assay, showing increased cell viability (Fig. 6C). Meanwhile, E8 supplemented with 1.5 μM CeONZs for 24 h during passage was able to reduce ROS to a level similar to Y27632 treated cells (Fig. 6D, E). When either Y27632 or CeONZs were added into E8 within 24 h, apoptosis was reduced after passaging (Fig. 6F and Additional file 1: Fig. S6A). In addition, when H9 hESCs were plated at clonal density (6000 cells/well in 6-well plate) on feeder layer in E8 supplied with Y27632, 1.5 μM, and 6 μM CeONZs, the highest number of total hESC colonies was observed in Y27632-treated cells on day 7, whereas untreated cells formed the fewest colonies and cells treated with 1.5 μM CeONZs produced intermediate number of colonies (Fig. 6G, H).

Next, we characterized H9 hESCs during continuous passaging using CeONZs. At the third passage, we also observed that CeONZs increased cell viability (Additional file 1: Fig. S6B). We then assessed the pluripotency of H9 hESCs up to the fifth generation (P5). The proportion of pluripotent stem cells marked by SSEA-4 expression in CeONZs-passaged P5 H9 hESCs was over 98% and the expression of pluripotency markers SOX2 and OCT4 was similar to Y27632-treated and non-treated H9 hESCs (Additional file 1: Fig. S6C, D). Furthermore, by performing qRT-PCR to detect the expression of pluripotency and lineage genes during passages 1, 3, and 5, we observed mild increase of pluripotency genes expression (*OCT4*, *SOX2*, *NANOG*, *KLF4*) in CeONZs-treated group compared to the cells cultured with E8 plus Y27623 or E8 only, while the expression of lineage markers, such as mesodermal (*TBXT*, *EOMES)*, endodermal (*HHEX*, *FOXA2*, *SOX17*, *AFP)*, ectodermal (*SOX1*, *SOX11*, *NESTIN*) genes remained at very low level (Additional file 1: Fig. S6E). Finally, we examined the spontaneous differentiation of hESCs passaged with CeONZs up to the fifth generation. Immunofluorescence staining revealed the presence of cells expressing endodermal (AFP), ectodermal (TUJ1), and mesodermal (FLK) markers (Additional file 1: Fig. S6F). All the above results suggested the protective role of CeONZs during hESCs maintenance without impairing pluripotency.

## Discussion

This study integrated nanotechnology with hPSCs to explore the potential application of CeONZs in stem cell-based cardiovascular disease therapy, focusing on hESCs and hESC-CMs. Our results proved that CeONZs showed high biocompatibility and minimal nanotoxicity to both hESCs and hESC-CMs across a wide range of concentrations. CeONZs reduced ROS levels in cells under oxidative stress, thereby improving cell viability. In a doxorubicin-induced human cardiotoxicity model, CeONZs mitigated oxidative stress and cellular injury by scavenging excessive ROS. Additionally, when introduced during hESCs passaging, CeONZs alleviated apoptosis by minimizing ROS production and enhancing cell survival. These findings supported the notion that CeONZs, acting as nanozymes, could protect hESCs and hESC-CMs from the detrimental effects of excessive ROS. This outcome may pave the way for CeONZs to serve as safe and effective as endogenous antioxidants, offering promising prospects for future clinical applications.

The XRD data confirmed that CeONZs have a cubic fluorite crystalline structure, with four characteristic diffraction peaks [68]. However, the TEM image showed an average core size of 5 ± 0.7 nm, larger than the crystalline size of 4.5 nm, possibly due to the particles being composed of one or more crystallites. The distance between lattice fringes was 0.34 nm which closely aligns with previously published literature (0.38 nm) [69]. The XPS peaks confirmed the presence of trivalent Ce^3+^ and tetravalent Ce^4+^, which contribute to the unique enzyme-mimicking activities and antioxidation properties of CeONZs [70]. Interestingly, under the optimal sonication conditions, the hydrodynamic size of CeONZs (~ 220 nm) was significantly larger than the original particle size, suggesting the presence of unbroken aggregates, which have been observed by other research groups as well [71]. The addition of H_2_O_2_ to the CeONZs solution oxidized and transformed the surface Ce^3+^ into Ce^4+^ through the CAT-mimicking activity, which was retrieved by boiling, ensuring the SOD enzyme mimicking activity. The SOD-mimetic enzyme activity of tested concentrations of CeONZs is in the range of physiological values in human body [40–42]. Such frequent reversibility between CAT and SOD mimicking activities is crucial for the ROS scavenging capabilities of CeONZs in vitro.

hESCs and hESC-CMs could quickly take up CeONZs upon their addition. Within the first 30 min, the cells engulfed 40% of the total amount of CeONZs that would be imported into the cells within 24 h. Previous studies have reported that human keratinocytes and cerebral microvascular endothelial cells also rapidly uptook a large amount of CeONZs in a short time, then gradually increased until reaching an equilibrium state [48, 72]. Subsequent investigations revealed that a significant proportion of CeONZs was translocated into mitochondria after cellular entry, similar to what was observed in human neuronal and endothelial cells [48, 73, 74]. Interestingly, we observed that the mitochondria in cardiomyocytes uptook 3 times more CeONZs compared to hESCs, possibly because of the larger number of mitochondria in hPSC-CMs than in hPSCs [75–77]. In the presence of ATP or caveolae protein inhibitor, the uptake of CeONZs by both H9 hESCs and H9-CMs was inhibited, suggesting an important role for endocytosis in the entry of CeONZs into the cell, as previously reported in other human cells [78, 79]. However, interestingly, when both pathway inhibitors were present, the cellular uptake of CeONZs was instead inferior to that of cells treated with the two pathway inhibitors individually. These results are likely due to crosstalk between different pathways [51] and cross-reactive nature of these inhibitors, as Genistein was reported to indirectly inhibit the uptake of 2-DG [80, 81]. The mechanisms underlying such crosstalk need to be revealed by further experiments in the future.

The effects of CeONZs on cells can vary depending on several factors, such as size, shape, surface chemistry, concentration, exposure time, and cell type [82, 83]. Our data revealed that CeONZs did not affect the proliferation and pluripotency of hESCs, as well as the contractile function and electrophysiology properties of hESC-CMs within the tested concentrations, indicating their good biocompatibility. This study expanded the potential application of CeONZs to hPSCs and their cardiac derivatives, providing crucial experimental evidence for their use in regenerative medicine.

As having been documented that CeONZs could shield H_2_O_2_-induced oxidative damage by ROS scavenging in other cell types [84–86], we further verified the ability of CeONZs to protect human cells from oxidative damage. In the two cellular stress models we adopted in this study, CeONZs displayed protective effects in dosage-independent manner. For post-treatment of CeONZs, higher concentration of H_2_O_2_ or TBHP (200 μM) treatment for 2 h severely reduced cell viability with stressed morphology (more than 50% decrease) (Fig. 4A, I, M). The overstressed cells may be not responsive to higher concentration of CeONZs (such as 6 μM) which may even exert additional stress on the cells. Meanwhile low concentration of CeONZs may be still uptaken by the stressed cells to facilitate their viability which was evidenced by partially reversal with 0.375 μM CeONZs in both H9 hESCs and H9-CMs. For co-treatment of CeONZs, lower concentration of H_2_O_2_ or TBHP (100 μM) treatment for 24 h without CeONZs resulted in relatively high cell viability (about or even less than 50% decrease) (Fig. 4C, K), in the presence of CeONZs, these less stressed cells may be more likely to uptake CeONZs at higher concentration (6 μM in these experiments) to protect the cells from stress damage. Thus, in the range of tested concentrations (0.375–6 μM) of CeONZs, the protective effects seem to be correlated with the cell status and stress models, with lower concentrations of CeONZs presenting better protective effects when cellular damage is high, and conversely, higher concentrations of CeONZs being more beneficial for less stressed cells. Further mechanistic interrogation is merited. In addition, the fact that CeONZs did not alter basal ROS levels in H9 and H9-CMs suggested that they exert their cell protection in the presence of excessive ROS. Our findings demonstrated that CeONZs improved cell activity by reducing ROS levels in both hESCs and hESC-CMs under oxidative stress.

Dexrazoxane (DXZ) is currently the only approved drug to prevent DOX-induced cardiotoxicity in cancer patients [87]. There is an urgent need to develop more effective alternatives for the prevention and treatment of DOX-induced cardiotoxicity. Relative mature differentiation day 120 cardiomyocytes were chosen for establishing the DOXIC model [61, 62]. Consistent with existing literature, DOX increased ROS, DNA damage, and decreased cell activity in hESC-CMs [88]. In contrast, CeONZs increased cardiomyocyte activity, decreased ROS, attenuated DNA damage, and increased anti-apoptotic gene expression to antagonize DOX-induced cardiotoxicity. We also observed that CeONZs reduced ROS and increased cell viability in differentiation day 30 cardiomyocytes. Interestingly, we found that 5 μM of DOX caused a 70% decrease in differentiation day 30 cardiomyocyte viability, while in differentiation day 120 cardiomyocytes the decrease was around 50%. Additionally, we found that 0.375 μM and 6 μM CeONZs provided optimal protection for differentiation day 30 and day 120 cardiomyocytes respectively. This variance in protective concentration of CeONZs was similar to what we observed in H_2_O_2_ or TBHP induced cellular stress models. The maturity of cardiomyocytes may be another factor contributed to the dosage-independent protective effects of CeONZs. While CeONZs have been reported to improve cardiac function in mice [89], our results provided further validation for the use of biocompatible CeONZs in human models, overcoming species differences and offering more robust evidence for their translational application.

Several chemical compounds have been verified, such as the ROCK inhibitor Y27632 [65] and the ‘CEPT’ molecule cocktails (Chroman 1, Emricasan, Polyamines, and Trans-ISRIB cocktails) [90] to improve the post-dissociation viability of hPSCs during passaging. Our results indicate that CeONZs can reduce the ROS levels induced by dissociation in hPSCs, and be safely used for consecutive cell passaging without compromising the stemness and differentiation potential of the cells. Of the two concentrations of CeONZs investigated, we observed that 1.5 μM of CeONZs supported H9 hESCs maintenance and colony formation, whereas 6 μM showed no significant impact. Y27632 has been routinely supplemented during hESCs passaging to enhance cell viability, as direct passaging with E8 only led to markedly low cell survival [65, 90]. Based on our findings in cellular stress models, 1.5 μM CeONZs might be more favorable to the cells than 6 μM in the case of severe cell damage. Furthermore, the variance in CeONZs uptake between suspended and adherent cells may have also contributed to these outcomes. In short, CeONZs posted an alternative cytoprotection strategy for the efficient and safe utilization of hPSCs.

Despite all these data, our study had certain limitations. Firstly, CeONZs exhibited dose-independent effects in mitigating cellular oxidative stress damage, indicating that the effective concentration of CeONZs may differ under varied oxidative stress conditions. Further research is warranted to elucidate the underlying mechanisms for this observation. Secondly, we focused on 2D models and did not extend the application of CeONZs to 3D models. As the advance of organoid research and single-cell technology, it would be valuable to supplement our study with more detailed investigations of CeONZs on cardioids, especially in-depth evaluation of CeONZs at the single-cell level, which would provide more comprehensive understanding of the bio- and nanosafety of CeONZs before clinical use. Finally, for effectively leveraging CeONZs in regenerative medicine, it is crucial to address the issue of targeted delivery, which remains a significant challenge.

## Conclusions

We successfully synthesized CeONZs which could be biosafely uptaken by hESCs and hESC-CMs to scavenge excessive oxidative stress-induced ROS, leading to improved cell viability. CeONZs significantly alleviated DOX-induced damage to human and rat cardiomyocytes and enhanced hESCs culture. These findings emphasized the potential application of CeONZs as a valuable resource in stem cell and regenerative medicine.

### Supplementary Information

**Additional file 1: Figure S1.** Characterization of CeONZs. **Figure S2.** Cardiomyocyte differentiation of hESCs. **Figure S3.** Biocompatibility of CeONZs in hESCs and hESC-CMs. **Figure S4.** Cell viability of hESC-CMs after exposure to different concentrations of H_2_O_2_ and TBHP. **Figure S5.** Protective effects of CeONZs on DOX-induced cardiotoxicity in H9-CMs and NRCMs. **Figure S6.** CeONZs increased the survival rate of H9 without affecting their stemness and differentiation potential. **Table S1.** Primary and Secondary Antibodies. **Table S2.** Primers used in qPCR experiments. **Table S3.** DNA damage of H_2_O_2_ and different concentrations of CeONZs treated H9 hESCs measured by comet test. **Table S4.** DNA damage of H_2_O_2_ and different concentrations of CeONZs treated H9-CMs measured by comet test.

## Data Availability

The data and materials used and analyzed for this work are available upon request to the corresponding author.
