# Supplementary material for: Biosafe cerium oxide nanozymes protect human pluripotent stem cells and cardiomyocytes from oxidative stress
Source: J Nanobiotechnology. 2024 Mar 26;22:132. doi: 10.1186/s12951-024-02383-x (PMC10967117; doi:10.1186/s12951-024-02383-x)
Supplement: Supplementary file 1 — Additional file 1: Figure S1. Characterization of CeONZs. Figure S2. Cardiomyocyte differentiation of hESCs. Figure S3. Biocompatibility of CeONZs in hESCs and hESC-CMs. Figure S4. Cell viability of hESC-CMs after exposure to different concentrations of H2O2 and TBHP. Figure S5. Protective effects of CeONZs on DOX-induced cardiotoxicity in H9-CMs and NRCMs. Figure S6. CeONZs increased the survival rate of H9 without affecting their stemness and differentiation potential. Table S1. Primary and Secondary Antibodies. Table S2. Primers used in qPCR experiments. Table S3. DNA damage of H2O2 and different concentrations of CeONZs treated H9 hESCs measured by comet test. Table S4. DNA damage of H2O2 and different concentrations of CeONZs treated H9-CMs measured by comet test. [file 12951_2024_2383_MOESM1_ESM.docx]

**Additional file 1**

**Biosafe Cerium Oxide Nanozymes Protect Human Pluripotent Stem Cells and Cardiomyocytes from Oxidative Stress**

Chengwen Hang^1,2,3,4┼^, Mohamed S Moawad^5┼*^, Zheyi Lin^1,2,3,4,6┼^, Huixin Guo^7^, Hui Xiong^1,2,3,4,8^, Mingshuai Zhang^1,2,3,4,8^, Renhong Lu^1,2,3,4^, Junyang Liu^1,2,3,4,8^, Dan Shi^1,2,3,4^, Duanyang Xie^1,2,3,4,6^, Yi Liu^1,2,3,4,6^, Dandan Liang^1,2,3,4,6,9^, Yi-Han Chen^1,2,3,4,6,9*^ Jian Yang^1,2,3,4,8,9*^

^1^State Key Laboratory of Cardiology, Shanghai East Hospital, Tongji University School of Medicine, Shanghai 200120, China. ^2^Shanghai Arrhythmia Research Center, Shanghai East Hospital, Tongji University School of Medicine, Shanghai 200120, China. ^3^Department of Cardiology, Shanghai East Hospital, Tongji University School of Medicine, Shanghai 200120, China. ^4^Shanghai Frontiers Center of Nanocatalytic Medicine, Shanghai 200092, China. ^5^Department of Toxicology and Forensic Medicine, Faculty of Veterinary Medicine, Cairo University, Giza 3725005, Egypt. ^6^Department of Pathology and Pathophysiology, Tongji University School of Medicine, Shanghai 200092, China. ^7^Department of Cardiology, The Second Hospital of Shanxi Medical University, Taiyuan 030001, China. ^8^Department of Cell Biology, Tongji University School of Medicine, Shanghai 200092, China. ^9^Research Units of Origin and Regulation of Heart Rhythm, Chinese Academy of Medical Sciences, Shanghai 200092, China.

^┼^Chengwen Hang, Mohamed S Moawad, and Zheyi Lin contributed equally to this work.

^*^Correspondence: Mohamed S Moawad; msalah@cu.edu.eg Yi-Han Chen; yihanchen@tongji.edu.cn Jian Yang; [jy279@tongji.edu.cn](mailto:jy279@tongji.edu.cn)


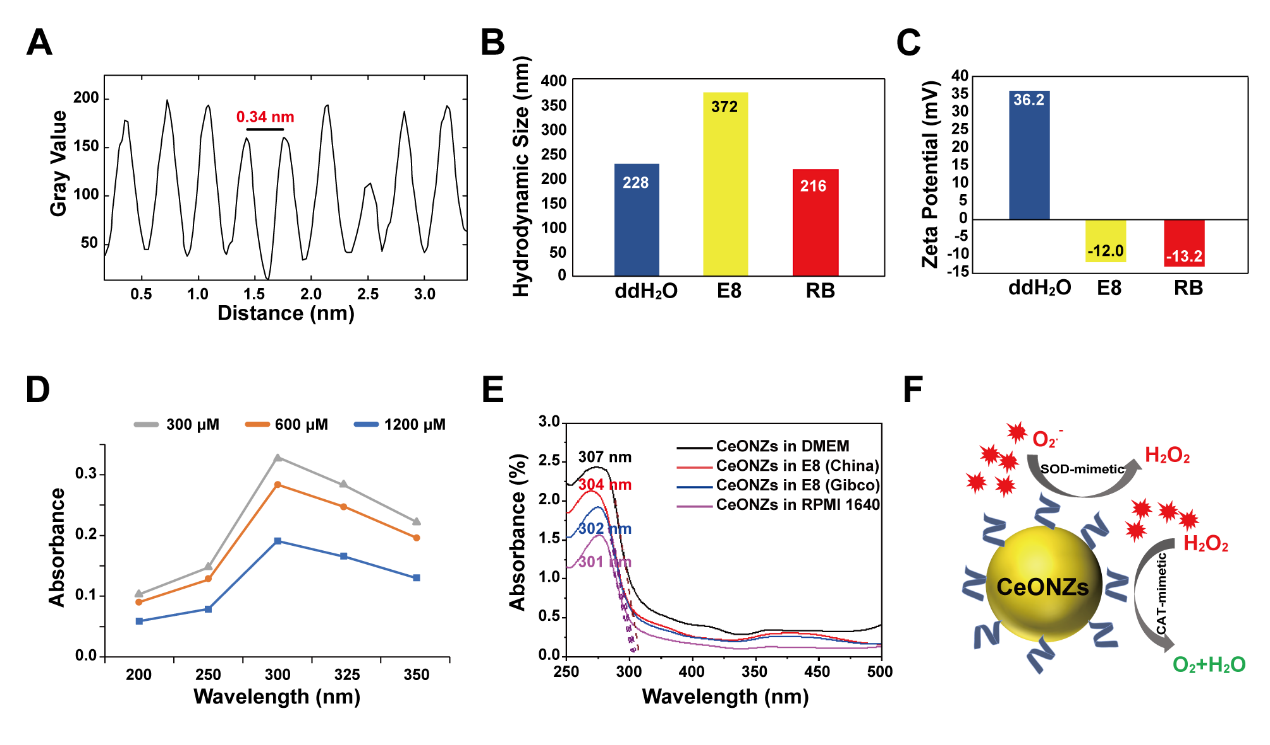


**Figure. S1** Characterization of CeONZs. **A** D-spacing of the characteristic (111) plane was plotted and calculated by ImageJ software. **B** Hydrodynamic sizes of CeONZs in ddH_2_O, E8, and RB. **C** Zeta potential of CeONZs in ddH_2_O, E8, and RB. **D** Absorption spectra of different concentrations of CeONZs in ddH_2_O measured by spectrophotometer. **E** Absorption spectra of CeONZs in DMEM, E8 (Cellapy), E8 (Gibco), and RPMI 1640. **F** The schematic diagram of CeONZs antioxidant enzyme activity.


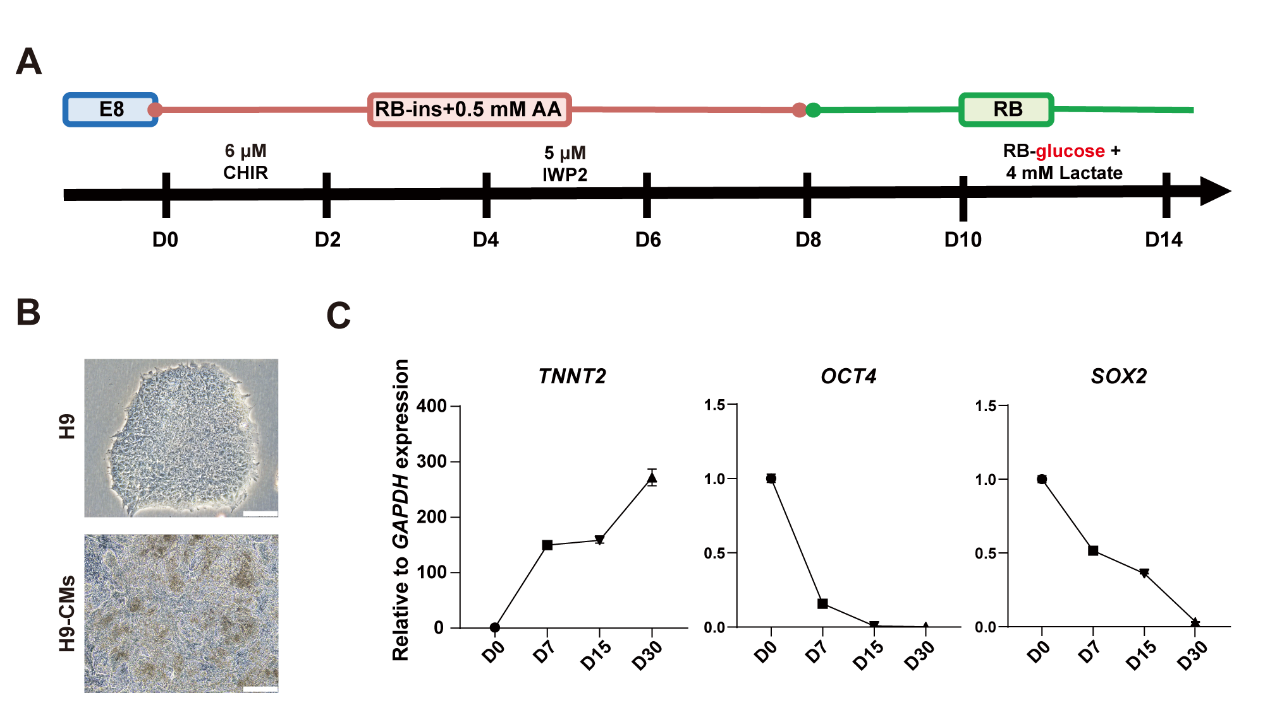


**Figure. S2** Cardiomyocyte differentiation of hESCs. **A** Schematic diagram of hESC-CMs generation. **B** Morphology of H9 hESCs and their derived cardiomyocytes (H9-CMs) (Scale bar = 100 μm). **C** Expression of CM marker *TNNT2* and pluripotency markers *OCT4* and *SOX2* at different days during cardiomyocyte differentiation. Relative to *GAPDH* expression (n = 3).


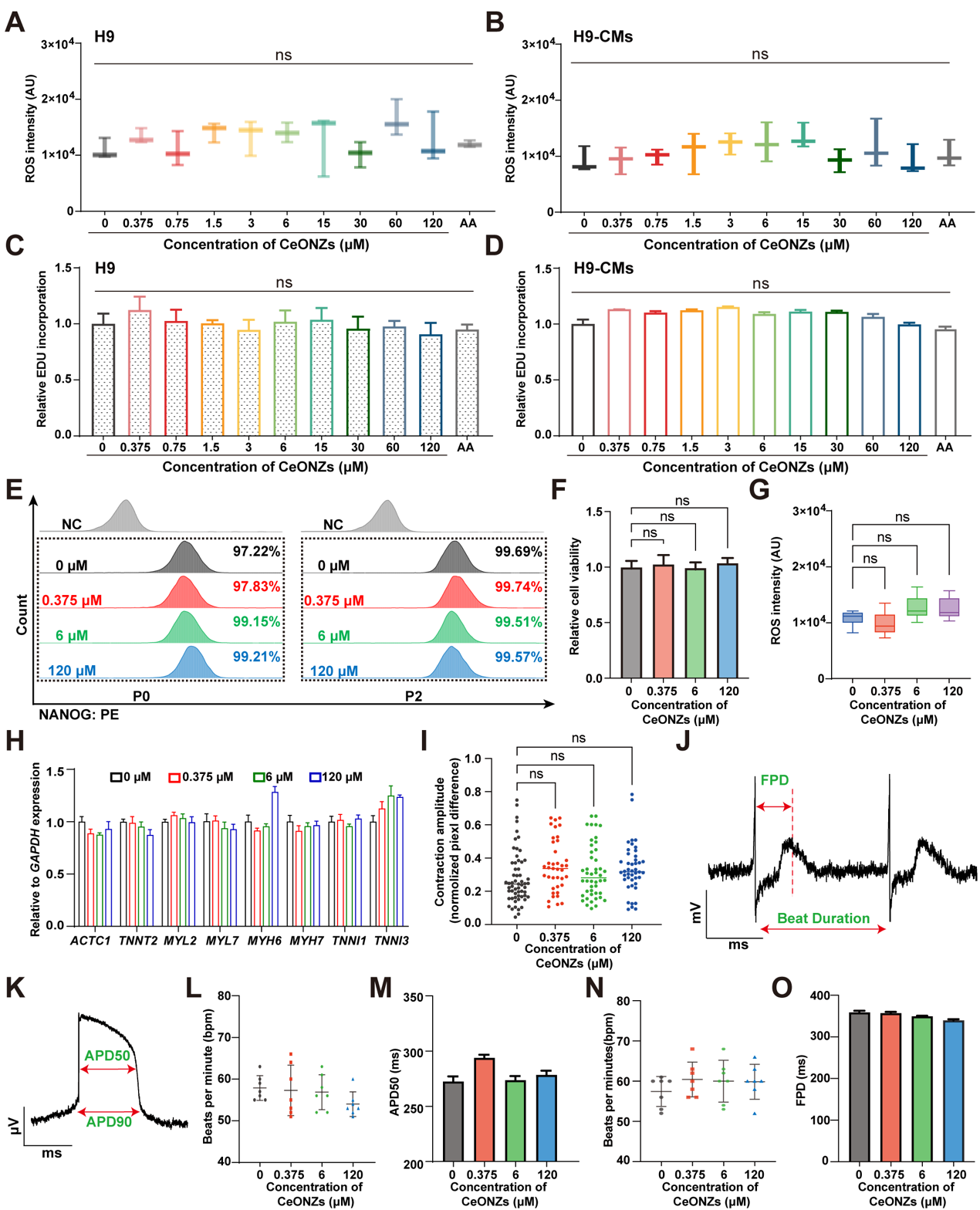


**Figure. S3** Biocompatibility of CeONZs in hESCs and hESC-CMs. **A** and **B** ROS intensity of H9 hESCs (**A**) and H9-CMs (**B**) treated with different concentrations of CeONZs and 0.5 mM AA for 24 h. **C** and **D** Relative EDU incorporation of H9 hESCs (**C**) and H9-CMs (**D**) treated with different concentrations of CeONZs and 0.5 mM AA for 24 h. **E** NANOG^+^ cells in H9 hESCs treated with low (0.375 μM), medium (6 μM), and high (120 μM) concentrations of CeONZs for 24 h (Passage 0, P0) and passaged 2 times after CeONZs withdrawal (Passage 2, P2). **F** and **G** Relative cell viability (**F**) and ROS intensity (**G**) of H9 hESCs treated with different concentrations of CeONZs at passage 2 (P2). **H** Gene expression analysis of myofilament-related genes (*ACTC1*, *TNNT2*, *MYL2*, *MYL7*, *MYH6*, *MYH7*, *TNNI1*, and *TNNI3*) in H9-CMs exposed to CeONZs for 24 h. **I** Contraction amplitude of H9-CMs after 24 h CeONZs exposure followed by culturing in basal medium for 3 days (n ≥ 39). **J** and **K** Schematic illustration of the output from MEA recording of field potentials (**J**) and action potentials (**K**). **L** and **M** Beat rate analysis (**L**) and action potential duration (APD50) (**M**) from MEA recording of H9-CMs treated with different concentrations of CeONZs for 24 h. **N** and **O** Beat rate analysis (**N**) and field potential duration (FPD) **(O)** of H9-CMs cultured in basal medium for 3 days after CeONZs removal. Data are shown as means ± SEM (n ≥ 3) and statistical significance was determined by one-way ANOVA with a Tukey post-test. ns means no significance.


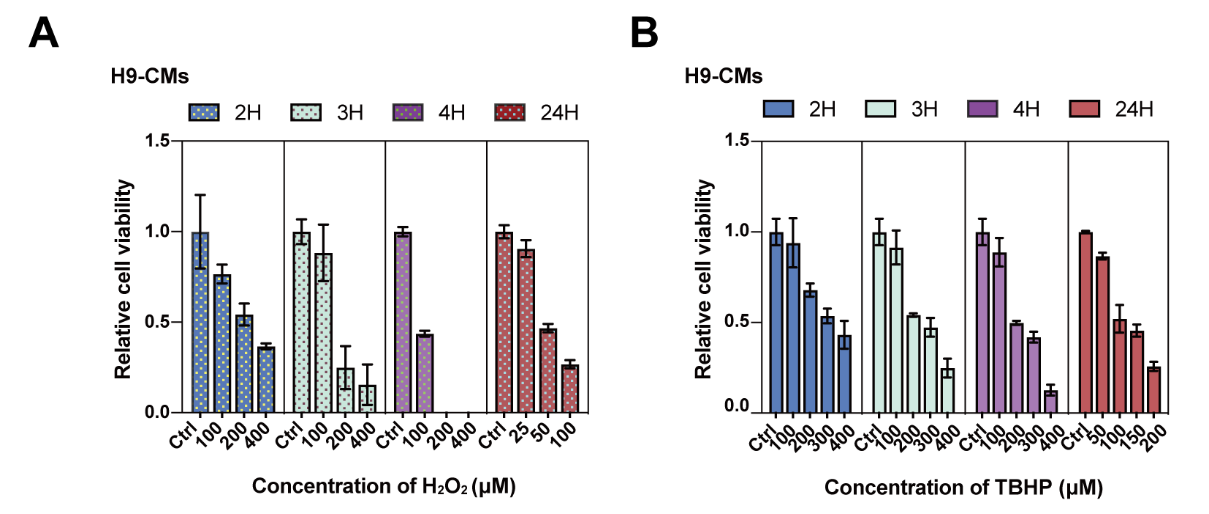


**Figure. S4** Cell viability of hESC-CMs after exposure to different concentrations of H_2_O_2_ and TBHP. **A** H9-CMs were incubated with indicated concentrations of H_2_O_2_ for 2 h, 3 h, 4 h, and 24 h, and cell viability was measured by CCK8 assay. **B** Cell viability of H9-CMs incubated with indicated concentrations of TBHP for the same time points and measured by CCK8 assay. Data are shown as means ± SEM (n ≥ 3).


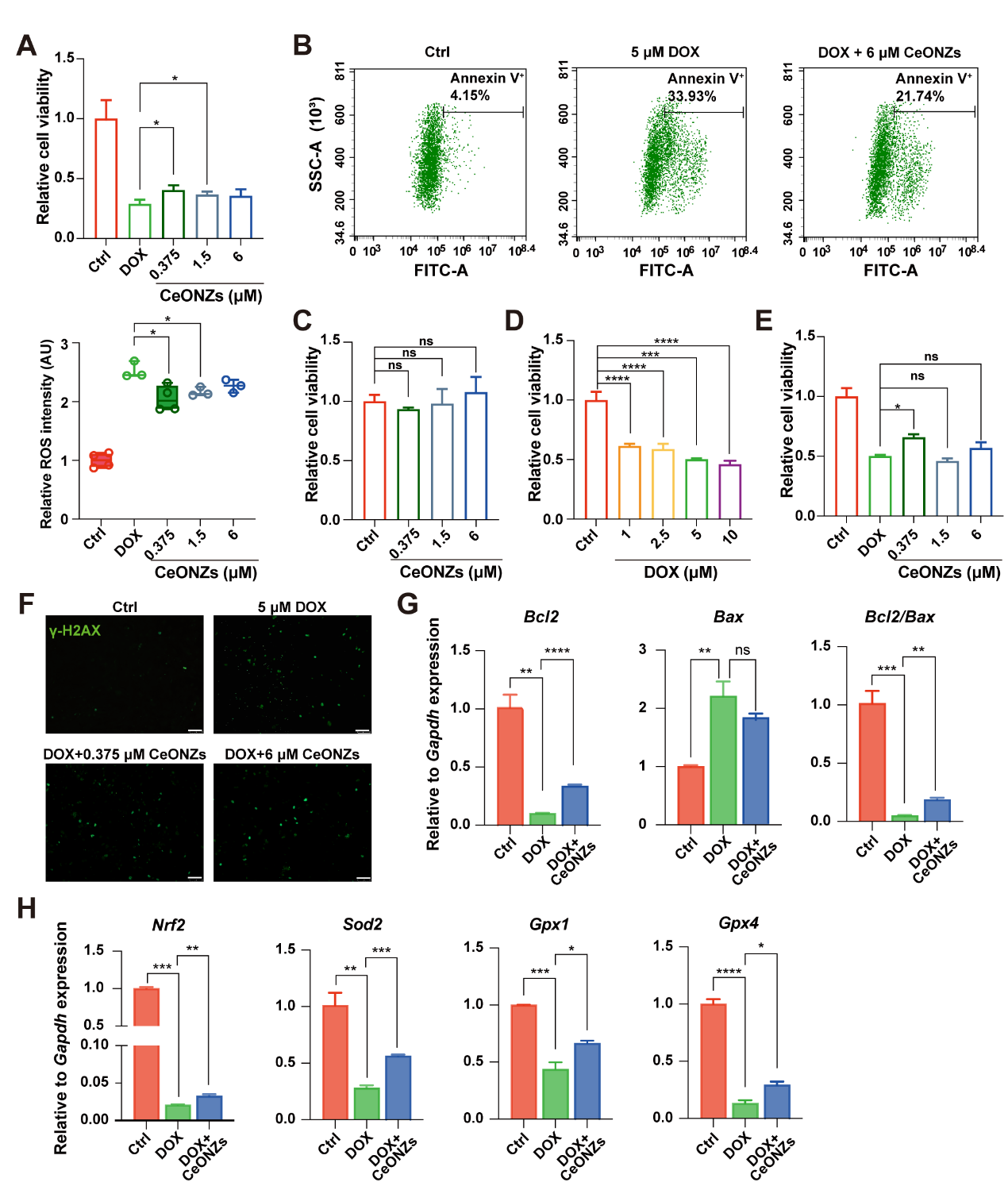


**Figure. S5** Protective effects of CeONZs on DOX-induced cardiotoxicity in H9-CMs and NRCMs. **A** Relative cell viability (top) and ROS intensity (bottom) in differentiation day 30 H9-CMs co-treated with 5 μM DOX with or without 0.375, 1.5, and 6 μM CeONZs. **B** The percentage of apoptotic population in H9-CMs treated with DOX and DOX plus 6 µM CeONZs. **C** Cell viability of neonatal rat cardiomyocytes (NRCMs) treated with 0.375, 1.5, and 6 μM CeONZs. **D** Cell viability of NRCMs exposed to different concentrations of DOX. **E** Cell viability of NRCMs treated with 5 μM DOX alone and in combination with 0.375, 1.5, and 6 μM CeONZs. **F** Immunofluorescence staining of DNA damage marker γ-H2AX in NRCMs treated with 5 μM DOX and 5 μM DOX with 0.375 and 6 µM CeONZs (Scale bar = 50 μm). **G** and **H** Analysis of apoptosis-related (**G**) and endogenous antioxidant-related (**H**) gene expression by qRT-PCR in NRCMs treated with 5 μM DOX and 5 μM DOX plus 0.375 μM CeONZs. Data are presented as means ± SEM (n ≥ 3), and statistical significance was determined by one-way ANOVA with a Tukey post-test. ns means no significance, * P < 0.05, ** P < 0.01, *** P < 0.001, **** P < 0.0001.


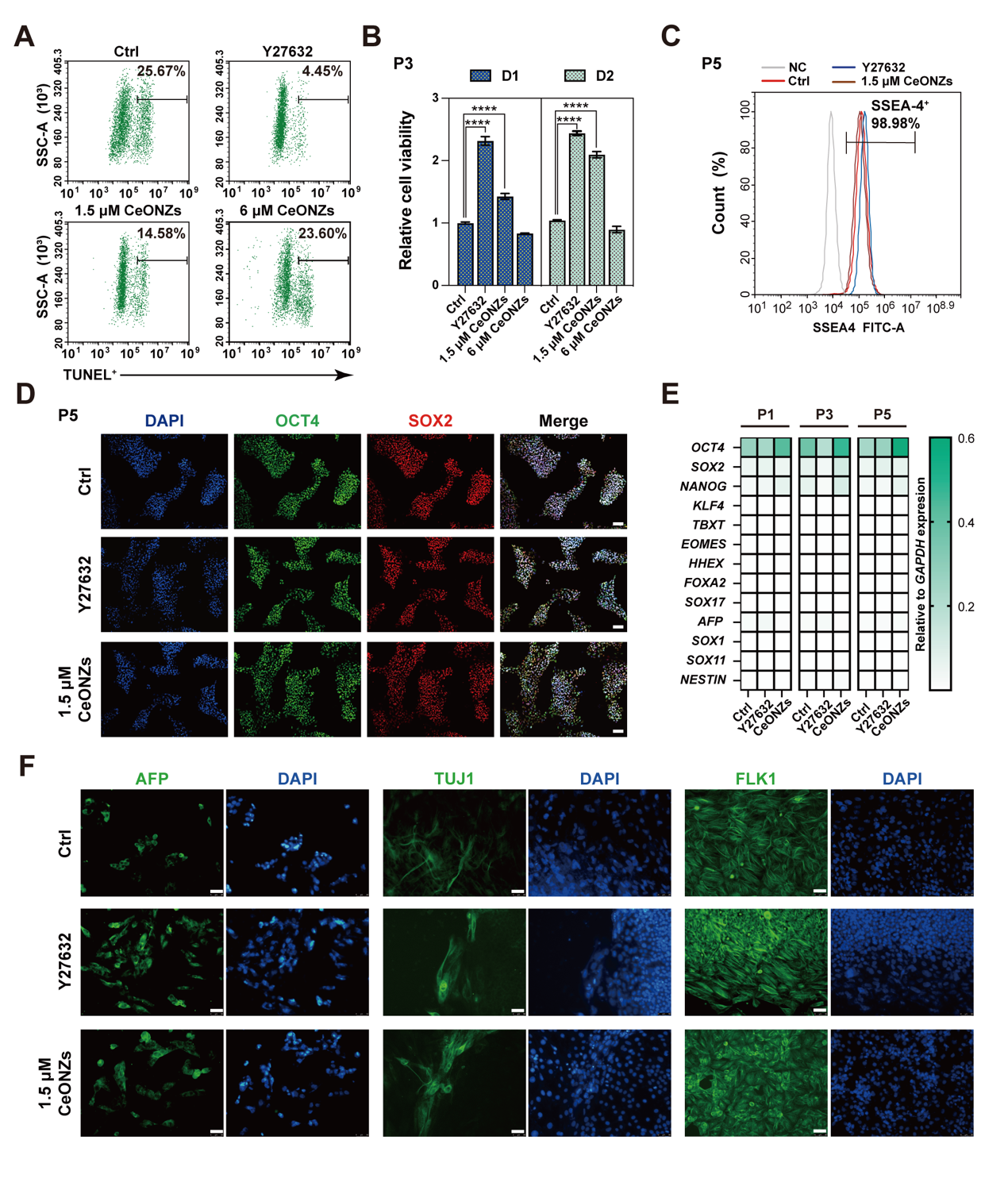


**Figure. S6** CeONZs increased the survival rate of H9 hESCs without affecting their stemness and differentiation potential. **A** Flow cytometry analysis of TUNEL^+^ populations of H9 hESCs passaged with E8 only, E8 plus Y27632, and E8 plus 1.5 μM or 6 μM CeONZs. **B** Cell viability of H9 hESCs after seeding 1 day and 2 days at passage 3. The cells were treated with E8 only, E8 plus Y27632, and E8 plus 1.5 μM, or 6 μM CeONZs for 24 h. **C** Expression of SSEA-4 in H9 hESCs passaged with E8 only, E8 plus Y27632, and E8 plus 1.5 μM CeONZs at the fifth passage by flow cytometry analysis. **D** Immunofluorescence staining of pluripotency markers OCT4 and SOX2 in H9 hESCs passaged with E8 only, E8 plus Y27632, and E8 plus 1.5 μM CeONZs at passage 5 (Scale bar = 100 μm). **E** Heatmap of pluripotency and lineage markers expression in H9 hESCs passaged with E8 only, E8 plus Y27632, and E8 plus 1.5 μM CeONZs at passage 1, 3, and 5. **F** Immunofluorescence of endodermal marker AFP, ectodermal marker beta-III Tubulin (TUJ1), and mesodermal marker FLK1 in EB differentiation of H9 hESCs passaged with E8 only, E8 plus Y27632, and E8 plus 1.5 μM CeONZs at the fifth passage (Scale bar = 50 μm). Data are presented as means ± SEM (n = 3), and statistical significance was determined by one-way ANOVA followed by Tukey’s post hoc test. ns means no significance, **** P < 0.0001.

Table S1. Primary and Secondary Antibodies

| Type | Antibody | Application | Dilution | Species | Manufacturer And Catalog Number |
| --- | --- | --- | --- | --- | --- |
| Primary | Anti-OCT4 | Immunofluorescence | 1:100 | Rabbit polyclonal | Abcam ab19857 |
|  | Anti-SSEA-4 | Immunofluorescence  Flow cytometry | 1:100 | Mouse  monoclonal | Sigma Aldrich  MAB4304 |
|  | Anti-NANOG | Flow cytometry | 1:100 | Rabbit polyclonal | Abcam  ab80892 |
|  | Anti-SOX2 | Immunofluorescence | 1:100 | Mouse  monoclonal | R&D system  MAB2018R |
|  | Anti-α-Actinin | Immunofluorescence | 1:100 | Rabbit polyclonal | Proteintech11313-2-AP |
|  | Anti-cTnT | Immunofluorescence  Flow cytometry | 1:100 | Mouse  monoclonal | Abcam ab19857 |
|  | Anti-AFP | Immunofluorescence | 1:50 | Mouse  monoclonal | Santa cruz  sc-8399 |
|  | Anti-TUJ1 | Immunofluorescence | 1:100 | Mouse  monoclonal | R&D system  MAB1195 |
|  | Anti-FLK1 | Immunofluorescence | 1:50 | Mouse  monoclonal | Santa Cruz  sc-393163 |
| Secondary | Goat Anti-Mouse IgG H&L (Alexa Fluor® 555) | Immunofluorescence | 1:200 | Goat Anti-Mouse | Abcam ab150114 |
|  | Goat Anti-Rabbit IgG H&L (Alexa Fluor® 488) | Immunofluorescence | 1:200 | Goat Anti-Rabbit | Abcam ab150077 |
|  | Goat Anti-Mouse IgG H&L (Alexa Fluor® 488) | Immunofluorescence | 1:200 | Goat Anti-Mouse | Abcam ab150113 |
|  | Goat Anti-Rabbit IgG H&L (Alexa Fluor® 555) | Immunofluorescence | 1:200 | Goat Anti-Rabbit | Abcam ab150078 |

**Table S2 Primers used in qPCR experiments.**

| **Primers used for Human cells (5’-3’)** | |
| --- | --- |
| h-*GAPDH*-F | GTCTCCTCTGACTTCAACAGCG |
| h-*GAPDH*-R | ACCACCCTGTTGCTGTAGCCAA |
| h-*OCT4*-F | CCTGAAGCAGAAGAGGATCACC |
| h-*OCT4*-R | AAAGCGGCAGATGGTCGTTTGG |
| h-*SOX2*-F | GCTACAGCATGATGCAGGACCA |
| h-*SOX2*-R | TCTGCGAGCTGGTCATGGAGTT |
| h-*NANOG*-F | CTCCAACATCCTGAACCTCAGC |
| h-*NANOG*-R | CGTCACACCATTGCTATTCTTCG |
| h-*KLF4*-F | CTCCCATCTTTCTCCACGTTC |
| h-*KLF4* -R | GTCGCTTCATGTGGGAGAG |
| h-*TNNT2*-F | GAGCAGACACCTCAAGTCCTG |
| h-*TNNT2*-R | CTCTACTGGACCATCTTCAGCA |
| h-*ACTC1*-F | TCTGGCTCCTAGCACCATGAAG |
| h-*ACTC1*-R | GCCTCATCGTACTCTTGCTTGC |
| h-*MYL2*-F | TACGTTCGGGAAATGCTGAC |
| h-*MYL2*-R | TTCTCCGTGGGTGATGATG |
| h-*MYL7*-F | CCGTCTTCCTCACGCTCTT |
| h-*MYL7*-R | TGAACTCATCCTTGTTCACCAC |
| h-*MYH6*-F | TCTCCGACAACGCCTATCAGTAC |
| h-*MYH6*-R | GTCACCTATGGCTGCAATGCT |
| h-*MYH7*-F | GGAGTTCACACGCCTCAAAGAG |
| h-*MYH7*-R | TCCTCAGCATCTGCCAGGTTGT |
| h-*TNNI1*-F | CGTGTGGACAAGGTGGATGAAG |
| h-*TNNI1*-R | GCCGCTTAAACTTGCCTCGAAG |
| h-*TNNI3*-F | CGTGTGGACAAGGTGGATGAAG |
| h-*TNNI3*-R | GCCGCTTAAACTTGCCTCGAAG |
| h-*TBXT*-F | AATTGGTCCAGCCTTGGAAT |
| h-*TBXT*-R | CGTTGCTCACAGACCACAG |
| h-*EOMES*-F | AAATGGGTGACCTGTGGCAAAGC |
| h-*EOMES*-R | CTCCTGTCTCATCCAGTGGGAA |
| h-*GSC*-F | GCACCATCTTCACTGACGAGCA |
| h-*GSC*-R | TTTGGCGCGGCGGTTCTTAAAC |
| h-*GATA4*-F | GCGGTGCTTCCAGCAACTCCA |
| h-*GATA4*-R | GACATCGCACTGACTGAGAACG |
| h-*HHEX*-F | CCAGGTGAGATTCTCCAACGAC |
| h-*HHEX*-R | CTCCATTTAGCGCGTCGATTCTG |
| h-*FOXA2*-F | ACTACCCCGGCTACGGTTC |
| h-*FOXA2*-R | AGGCCCGTTTTGTTCGTGA |
| h-*SOX17*-F | GGGGACATGAAGGTGAAGGG |
| h-*SOX17-*R | GTGCAGGTCTGGATTCTGCT |
| h-*AFP*-F | GCAGAGGAGATGTGCTGGATTG |
| h-*AFP-* R | CGTGGTCAGTTTGCAGCATTCTG |
| h-*SOX1*-F | GAGTGGAAGGTCATGTCCGAGG |
| h-*SOX1*-R | CCTTCTTGAGCAGCGTCTTGGT |
| h-*SOX11*-F | GCTGAAGGACAGCGAGAAGATC |
| h-*SOX11*-R | GGGTCCATTTTGGGCTTTTTCCG |
| h-*NESTIN*-F | GGAGAAGGACCAAGAACTG |
| h-*NESTIN*-R | ACCTCCTCTGTGGCATTC |
| h-*TUBB3*-F | TCAGCGTCTACTACAACGAGGC |
| h-*TUBB3*-R | GCCTGAAGAGATGTCCAAAGGC |
| h-*BAX*-F | TCAGGATGCGTCCACCAAGAAG |
| h-*BAX-*R | TGTGTCCACGGCGGCAATCATC |
| **Primers used for Rat cells (5’-3’)** | |
| R-*Bcl2*-F | CTTTGAGTTCGGTGGGGTCA |
| R-*Bcl2*-R | TAGTTCCACAAAGGCATCCCAG |
| R-*Bax*-F | CGTCTGCGGGGAGTCAC |
| R-*Bax*-R | CGATCCTGGATGAAACCCTGT |
| R-*Nrf2*-F | TGTCAGCTACTCCCAGGTTG |
| R-*Nrf*2-R | AATATCCAGGGCAAGCGAC |
| R-*Sod2*-F | CGGGGGCCATATCAATCACA |
| R- *Sod2*-R | CCAGCAACTCTCCTTTGGGT |
| R-*Gpx1*-F | TCAGTTCGGACATCAGGAGAAT |
| R-*Gpx1*-R | TCACCATTCACCTCGCACTT |
| R-*Gpx4*-F | GAGCCCATTCCCGAGCCTTT |
| R-*Gpx4*-R | CGCGGGATGCACACAAGC |

**Table S3 DNA damage of H_2_O_2_ and different concentrations of CeONZs treated H9 hESCs measured by comet test**

|  | **200μM H_2_O_2_ treated** | **Concentration of CeONZs** | | | |
| --- | --- | --- | --- | --- | --- |
|  |  | **0 μM** | **0.375 μM** | **6 μM** | **120 μM** |
| **Tail Length (pixels)** | 194.3 ± 76.8 | 18.4 ± 25.4 | 17.5 ± 22.3 | 21.7 ± 39.4 | 38.4 ± 49 |
| **Tail DNA (%)** | 82 ±10.4 | 13.0 ± 25.3 | 10.4 ± 22.4 | 9 ± 19.7 | 19.6 ± 31.9 |
| **Tail moment (μm)** | 33.8 ± 76.1 | 7.4 ± 44.7 | 5.5 ± 19.1 | 8.6 ± 35.1 | 20.5 ± 43.8 |
| **Olive Tail moment** | 82.3 ± 33.2 | 6.0 ±15.4 | 4.7 ±11.3 | 7.2 ±24.2 | 13.1 ± 24.6 |

Data are presented as Mean ± SD.

**Table S4 DNA damage of H_2_O_2_ and different concentrations of CeONZs treated H9-CMs measured by comet test**

|  | **200μM H_2_O_2_ treated** | **Concentration of CeONZs** | | | |
| --- | --- | --- | --- | --- | --- |
|  |  | **0 μM** | **0.375 μM** | **6 μM** | **120 μM** |
| **Tail Length (pixels)** | 104.1 ± 50.6 | 23.3 ± 28 | 13 ± 18.9 | 18.9 ± 22.1 | 16.7 ± 25.3 |
| **Tail DNA (%)** | 81.7 ± 28.1 | 34.2 ± 38.7 | 21.3 ± 32.9 | 29.2 ± 35.6 | 22.6 ± 33.8 |
| **Tail moment (μm)** | 73.1 ± 35 | 5.1 ± 16.9 | 0.8 ± 5.8 | 1.7 ± 7.8 | 2.1 ± 11.5 |
| **Olive Tail moment** | 53.1 ± 23.6 | 5.5 ± 13.1 | 2.5 ± 10.1 | 6.7 ±14.3 | 2.4 ± 10.5 |

Data are presented as Mean ± SD.
